# Prevalence and risk factors for milk allergy overdiagnosis in the BEEP trial cohort

**DOI:** 10.1111/all.16203

**Published:** 2024-06-20

**Authors:** Hilary I. Allen, Olivia Wing, Dara Milkova, Emilia Jackson, Karen Li, Lucy E. Bradshaw, Laura Wyatt, Rachel Haines, Miriam Santer, Andrew W. Murphy, Sara J. Brown, Maeve Kelleher, Michael R. Perkin, Nicola Jay, Timothy D. H. Smith, Frank Moriarty, Alan A. Montgomery, Hywel C. Williams, Robert J. Boyle

**Affiliations:** ^1^ National Heart and Lung Institute Imperial College London London UK; ^2^ Centre of Evidence Based Dermatology, Lifespan and Population Health University of Nottingham Nottingham UK; ^3^ Nottingham Clinical Trials Unit, School of Medicine University of Nottingham Nottingham UK; ^4^ Primary Care Research Centre University of Southampton Southampton UK; ^5^ Department of General Practice & HRB Clinical Trial Network Primary Care Ireland University of Galway Galway Ireland; ^6^ Centre for Genomic and Experimental Medicine University of Edinburgh Edinburgh UK; ^7^ Children's Health Ireland Crumlin Children's Hospital Dublin Ireland; ^8^ Population Health Research Institute St George's University of London London UK; ^9^ Sheffield Children's NHS Foundation Trust Sheffield UK; ^10^ NIHR Clinical Research Network North West Coast Primary Care Team Liverpool UK; ^11^ School of Pharmacy and Biomolecular Sciences RCSI University of Medicine and Health Sciences Dublin Ireland

**Keywords:** cow's milk allergy, low‐allergy formula, overdiagnosis, primary care

## Abstract

**Background:**

Cow's milk allergy (CMA) overdiagnosis in young children appears to be increasing and has not been well characterised. We used a clinical trial population to characterise CMA overdiagnosis and identify individual‐level and primary care practice‐level risk factors.

**Methods:**

We analysed data from 1394 children born in England in 2014–2016 (BEEP trial, ISRCTN21528841). Participants underwent formal CMA diagnosis at ≤2 years. CMA overdiagnosis was defined in three separate ways: parent‐reported milk reaction; primary care record of milk hypersensitivity symptoms; and primary care record of low‐allergy formula prescription.

**Results:**

CMA was formally diagnosed in 19 (1.4%) participants. CMA overdiagnosis was common: 16.1% had parent‐reported cow's milk hypersensitivity, 11.3% primary care recorded milk hypersensitivity and 8.7% had low‐allergy formula prescription. Symptoms attributed to cow's milk hypersensitivity in participants without CMA were commonly gastrointestinal and reported from a median age of 49 days. Low‐allergy formula prescriptions in participants without CMA lasted a median of 10 months (interquartile range 1, 16); the estimated volume consumed was a median of 272 litres (26, 448). Risk factors for CMA overdiagnosis were high practice‐based low‐allergy formula prescribing in the previous year and maternal report of antibiotic prescription during pregnancy. Exclusive formula feeding from birth was associated with increased low‐allergy formula prescription. There was no evidence that practice prescribing of paediatric adrenaline auto‐injectors or anti‐reflux medications, or maternal features such as anxiety, age, parity and socioeconomic status were associated with CMA overdiagnosis.

**Conclusion:**

CMA overdiagnosis is common in early infancy. Risk factors include high primary care practice‐based low‐allergy formula prescribing and maternal report of antibiotic prescription during pregnancy.

## Graphical abstract

In BEEP, cow's milk hypersensitivity was commonly reported in infants without confirmed milk allergy. One in six parents reported non‐confirmed cow's milk hypersensitivity in their child, and half of these were prescribed low‐allergy formula during the first year. The strongest risk factor for milk allergy overdiagnosis was high primary care practice‐based prescribing of low‐allergy formula in the year before birth. Abbreviations: BEEP, Barrier Enhancement for Eczema Prevention; CMA, cow's milk allergy

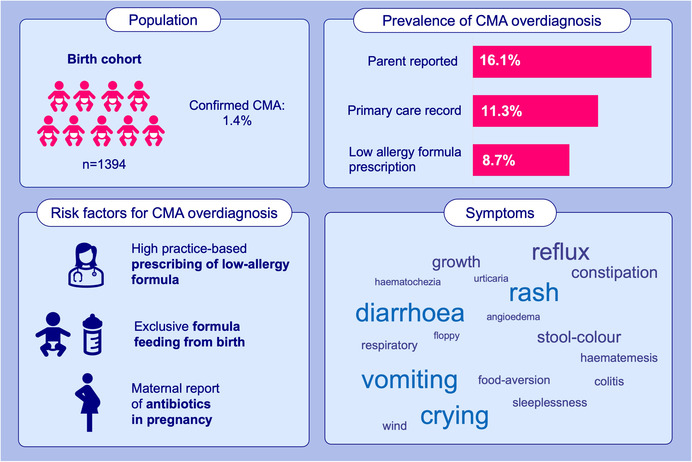

## 1 INTRODUCTION

Cow's milk allergy (CMA) affects about 1% of children under 2 years.^1^ In UK, USA, Norway and Australia, prescription rates of specialised low‐allergy formula are up to 15 times higher than expected, suggesting CMA overdiagnosis.^2^, ^3^ The consequences of unnecessary exposure of large numbers of non‐allergic infants to prescription formula designed to manage CMA are unknown.^3^, ^4^, ^5^, ^6^ Low‐allergy formulas partially or completely substitute lactose with alternative carbohydrate sources, such as glucose syrup and maltodextrin, and these ‘free sugars’ may carry risks to child health and development.^3^, ^6^, ^7^, ^8^, ^9^ The World Health Organisation and other public health bodies recommend limiting exposure to free sugars due to concerns about obesity and dental health.^4^, ^10^ Glucose syrup‐based infant formula provision was associated with increased early childhood obesity in the United States.^7^ Other potential consequences of CMA overdiagnosis include resource waste, maternal psychological distress and early cessation of breastfeeding.^11^

Milk allergy overdiagnosis has not been well‐characterised and appears to be increasing worldwide^2^, ^3^, ^12^ In this study, we used a clinical trial birth cohort with a prospective evaluation of CMA diagnosis to describe features of CMA overdiagnosis and explore potential risk factors.^13^

## 2 METHODS

### 2.1 Study design

Retrospective analysis of primary care records for children with a parent‐reported milk reaction during participation in the Barrier Enhancement for Eczema Protection (BEEP) clinical trial.^13^ BEEP was a prospective, community‐based randomised clinical trial of a skincare intervention in 1394 infants enrolled at birth in England (2014–2016). Primary care records were requested from practices of BEEP study participants whose parents reported a reaction to cow's milk at 12‐ or 24‐month questionnaires and who did not opt out of this primary care record evaluation. Ethical approval was granted by the West Midlands Ethics Committee (14/WM/0162).

### 2.2 Cow's milk allergy diagnosis

Participants had a family history of atopic disease and were assessed for milk allergy at ages 12 and 24 months. Three screening questions were: ‘in the last year, has your baby had a reaction to any foods containing cow's milk protein?’ (12 months), ‘has your child had a reaction to foods containing cow's milk?’ (24 months) and ‘in the last year, has your child been prescribed special low allergy formula milk?’ (24 months). Children whose parents answered yes to any screening question underwent formal diagnostic assessment for IgE‐mediated CMA at age 2 years as part of the BEEP study, with skin prick testing, clinical history and oral food challenge or expert panel review.^14^ For this analysis, we also identified additional cases of IgE‐mediated CMA that resolved prior to the age of 2 years, and non‐IgE‐mediated CMA confirmed by formal oral food challenge or elimination and re‐introduction through review of BEEP trial records and primary care records. Participants who did not answer yes to any screening question were considered not to have CMA and those who did not answer any screening question were considered non‐responders.

### 2.3 Cow's milk allergy overdiagnosis

CMA overdiagnosis was defined in three ways, each analysed separately. Definitions were parent‐reported milk reaction, categorised using the three screening questions; primary care record of milk hypersensitivity symptoms; and primary care record of low‐allergy formula prescription. Participants with confirmed CMA diagnosis were excluded from all three definitions.

### 2.4 Primary care record data collection

Primary care records, including consultation notes, prescriptions and correspondence, were analysed independently by three investigators (HA, DM and EJ). Data collected included primary care record of milk reaction and prescription of specialised low‐allergy formula (extensively hydrolysed, amino‐acid or soya formula, as defined elsewhere^2^; see Appendix S1).

### 2.5 Practice‐level data collection

Practice‐level prescribing data for practices in England in 2014 were extracted for specialised low‐allergy formula, junior adrenaline auto‐injectors (AAI) and anti‐reflux medications used in infants and young children. Data were extracted from NHS Business Service Authorities (NHSBSA) using R code (Appendix S1).^15^, ^16^, ^17^ Data for 2014, prior to birth of the first BEEP study participants, were chosen to ensure BEEP participant prescribing data were not included. Data were linked to individual BEEP participant practice codes. Total quantity (grams) of low‐allergy formula was converted to volume (litres) using the British National Formulary for children (BNFc) weight‐to‐volume conversion rates.^18^ AAI quantity was determined by number of items prescribed. Specific anti‐reflux medications, formulations and doses used for managing reflux symptoms in infants were identified through a survey of primary and secondary care practitioners with an interest in allergy and gastroenterology, and the total items prescribed was calculated. Since most of these anti‐reflux medications are also used beyond the first 2 years of life, we separately analysed quantity of Gaviscon® infant alginate sachets prescribed. Gaviscon® infant is only indicated for use in the first 2 years of life (Table S1). Practice antibiotic prescribing data were extracted from the NHSBSA Catalyst public database^19^ as an indicator of practice over‐prescribing, based on previous evidence that antibiotics are over‐prescribed in primary care.^20^, ^21^ Total antibiotic items and antibiotic items per Specific Therapeutic group Age‐Sex Related Prescribing Unit (STAR‐PU) were recorded from Catalyst. STAR‐PU is an indicator which adjusts for age and gender distribution within a practice population for antibiotic prescribing.^19^, ^22^, ^23^

Other practice‐level data extracted were a decile of the English index of multiple deprivation 2019 based on the primary care practice postcode; practice demographics from the NHS Digital patient registry; Clinical Commissioning Group (CCG) characteristics from the Office for National Statistics database; and a categorisation of local CCG milk allergy guideline recommendations in relation to a recent Delphi consensus study (Table S2).^24^, ^25^, ^26^, ^27^, ^28^

### 2.6 Statistical analysis

Analyses were performed using Statistical Package for Social Sciences (SPSS version 29, IBM; Appendix S1).^29^ Visual inspection of histograms was used to assess normality of data distribution. Backward logistic regression was used to explore associations between participant‐level risk factors and CMA overdiagnosis. Mixed‐effects logistic regression with complete case analysis was used to assess practice‐level risk factors and adjust for the clustering of participants within practices. Sensitivity analyses were conducted by substituting antibiotic items for antibiotic items/STAR‐PU; using multiple imputations to account for missing data; and assessing amino acid formula (AAF) alone as trends in volume prescribed differed over time compared to extensively hydrolysed formula (EHF). AAF also differs from EHF in carbohydrate and protein content and the impact on health may not be the same. Statistical tests for significance between confirmed CMA and CMA overdiagnosis included the Mann–Whitney *U* test (non‐parametric data), chi‐squared and Fisher's exact test (categorical data) and Benjamini–Hochberg method to control the false discovery rate at 5%.^29^, ^30^

## 3 RESULTS

**FIGURE 1:**
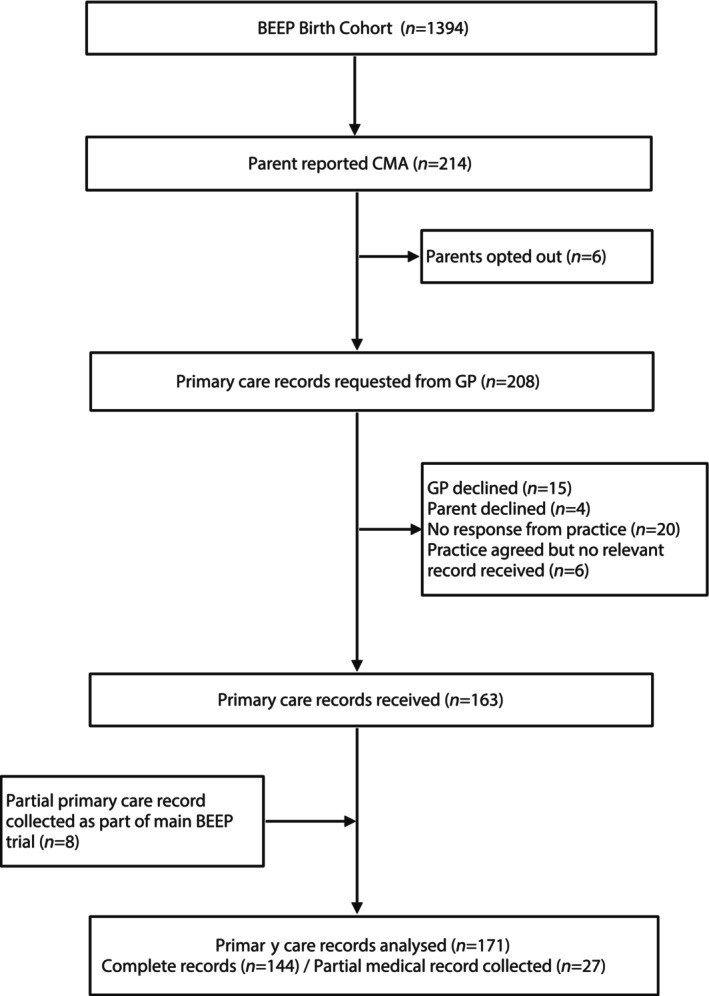
Flowchart of data collection. Primary care records were requested for 208/1394 (14.9%) BEEP participants. Of these, 171/1394 (12.3%) were successfully analysed for a reaction to cow's milk and/or low‐allergy formula prescription. BEEP, Barrier Enhancement for Eczema Prevention; GP, General Practitioner. Although only six participants opted out of the primary care record review, a further four opted out after the primary care practitioner (general practitioner, GP) independently contacted the family to confirm their consent for analysis of the child's primary care records.

Data collection is summarised in Figure 1. In BEEP, 214 participants reported a milk reaction and/or low‐allergy formula prescription. Primary care records were successfully obtained and analysed for 171/214 (80%) of these. Nineteen of 214 had confirmed CMA, 18 IgE‐mediated and 1 non‐IgE mediated (Table S3).^13^

### 3.1 Incidence of CMA overdiagnosis

We estimated 16.1% of children without CMA in BEEP had a parent‐reported milk reaction by age 2 years, with 11.3% having a primary care record of milk hypersensitivity and 8.7% prescribed low‐allergy formula (Table 1, Figure S1). At age 12 months, 105 participants reported a milk reaction, 80% of whom had documented cow's milk hypersensitivity (Table S4). Similarly, 85% of participants who reported a milk reaction at 24 months had a documented primary care record of cow's milk hypersensitivity (Table S4). While 94% of participants who reported low‐allergy formula use had ≥1 documented prescription, 36% of those reporting a milk reaction but no low‐allergy formula use also had a documented prescription (Table S4).

**TABLE 1:** Prevalence of cow's milk hypersensitivity and low‐allergy formula prescription in BEEP study cohort.

	Total BEEP cohort		BEEP excluding participants with confirmed CMA	
	Documented rate *n*/*N* (%)	Estimated rate *n*/*N* (%)	Documented rate *n*/*N* (%)	Estimated rate *n*/*N* (%)
Parent report of milk reaction	214/1394 (15.4%)	243/1394 (17.4%)	195/1375 (14.2%)	222/1375 (16.1%)
Primary care record of milk hypersensitivity	140/1394 (10.0%)	175/1394 (12.6%)	124/1375 (9.0%)	156/1375 (11.3%)
Low‐allergy formula prescription	91/1394 (6.5%)	133/1394 (9.6%)	81/1375 (5.9%)	119/1375 (8.7%)

*Note*: Cow's milk hypersensitivity refers to any concern about hypersensitivity to cow's milk. Low‐allergy formula prescription includes extensively hydrolysed, amino acid and soya formula. Documented rate is the number of identified cases in the available records and assumes all other participants did not have reported cow's milk hypersensitivity or low‐allergy formula prescription. Estimated rate assumes the same proportion of parent‐reported milk reactions, primary care records of cow's milk hypersensitivity or primary care records of low‐allergy formula prescription in the unavailable records. Of 91 participants who were prescribed low‐allergy formula, number of prescriptions was available for 72 participants, of which 14 (19%) were one‐off prescriptions and the others had repeat prescriptions.

### 3.2 Timing of CMA overdiagnosis

Median age at documented symptom onset and first primary care record documentation of milk hypersensitivity diagnosis was 49 days (IQR 34, 160) and 163 days (61, 284) for participants CMA overdiagnosis, and 102 days (47, 184) and 181 days (125, 249) for participants with confirmed CMA (Figure S2). Median time between documented symptom onset and diagnosis was 37 days (IQR 14, 91) for CMA overdiagnosis and 56 days (39, 109) for confirmed CMA.

Timing (measured as age of child) of first mention of maternal dietary exclusion and first dietetic review is shown in Figure S3, for participants who had timing of maternal dietary exclusion or dietetic review documented in primary care records. Maternal dietary restriction advice was documented earlier in CMA overdiagnosis (median 76 days, IQR 45, 156) than in confirmed CMA (median 156 days, 135, 236; *p* = .007). First dietitian review occurred at median 261 days (IQR 159, 399) in CMA overdiagnosis and 350 days (214, 435) in confirmed CMA. Timing of symptom onset, diagnosis and dietitian review were all earlier in CMA overdiagnosis than in confirmed CMA, but differences were not statistically significant.

Timing of low‐allergy formula prescription is shown in Figures S4 and S5, for the subset of 83 participants where timing was clearly documented in the primary care record. First formula prescription occurred at a median of 121 days (IQR 57, 225) in CMA overdiagnosis and 139 days (95, 283) in confirmed CMA. Final prescription occurred at a median of 429 days (304, 633) in CMA overdiagnosis and 388 days (318, 576) in confirmed CMA. There were no significant differences between the two groups in these timings.

### 3.3 Characteristics of CMA overdiagnosis

**FIGURE 2:**
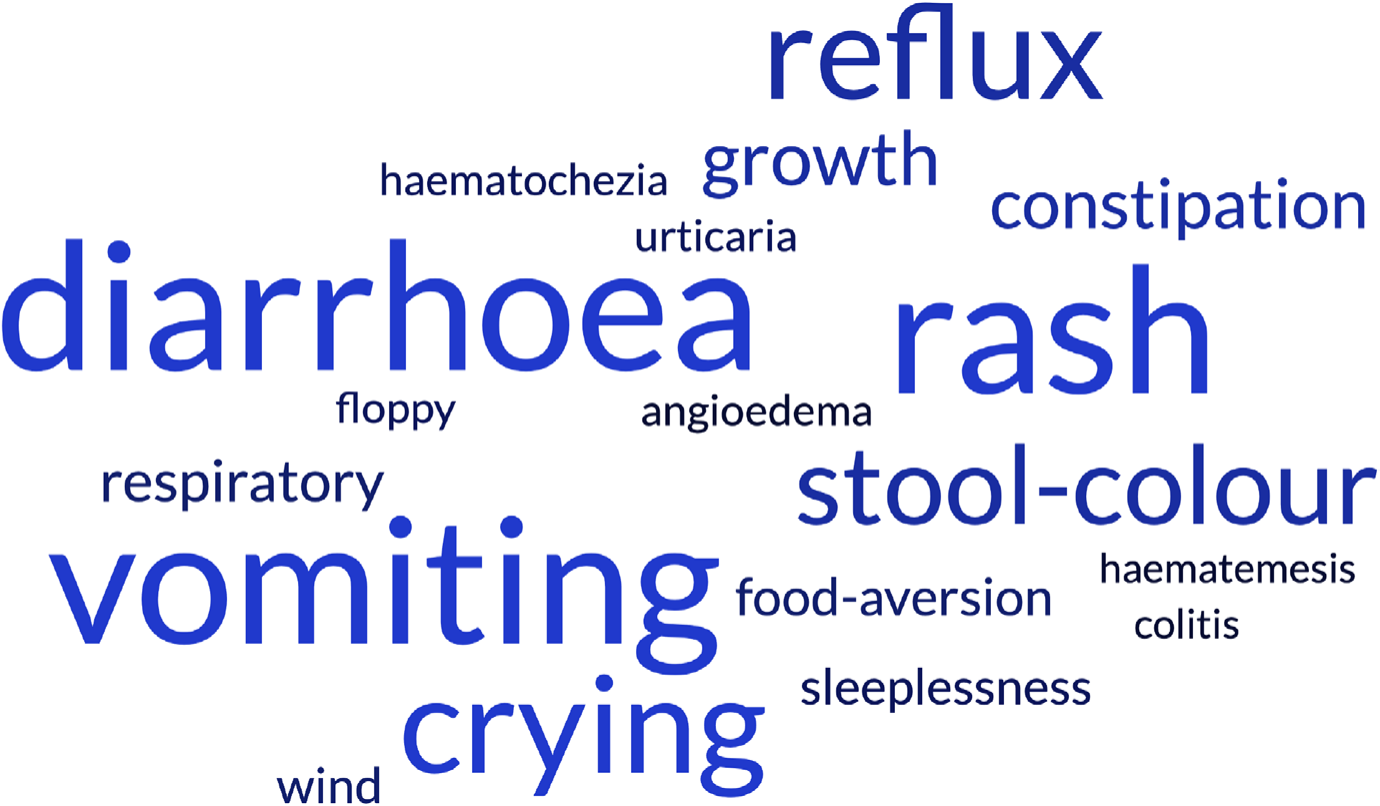
Symptoms recorded at the time of first reaction to milk in children with CMA overdiagnosis. Symptoms recorded in the primary care record at time of first mention of a reaction to cow's milk in children who did not have confirmed CMA. Size of words represents the frequency of individual symptoms leading to a diagnosis of possible milk reaction. Word cloud was generated using https://www.freewordcloudgenerator.com.

Characteristics of CMA overdiagnosis in BEEP are summarised in Table S5. Primary care records suggest the possibility of cow's milk hypersensitivity was most commonly raised by primary care physicians (General Practitioner, GP), but in >20% parents raised the initial concern. Definitive clinical diagnosis was most frequently given in secondary care, with ‘allergy’ as the most common diagnostic label. Symptoms were most commonly lower gastrointestinal (58% (72/124)) (Figure 2), and skin symptoms were less common than in confirmed CMA (40% vs. 94%, *p* < .001, adjusted for false discovery *p* = .006; Figure S6).^30^ For most cases, no formal diagnostic process was undertaken, and where undertaken, test results were usually negative (23/29, 79%). In contrast, for confirmed CMA, tests were usually positive (8/10, 80%), when undertaken. At the time when concern about cow's milk hypersensitivity was first documented, 43% of those with CMA overdiagnosis and 75% confirmed CMA were partially or fully breastfed, and rates were similar at the time of first low‐allergy formula prescription (Table S6). Most participants with CMA overdiagnosis (66%) or confirmed CMA (75%) had maternal dietary restriction of dairy, usually parent‐initiated; although in almost half, a healthcare practitioner also suggested maternal dietary restriction. Most CMA overdiagnosis or confirmed CMA participants were referred for dietetic review.

### 3.4 Patterns of low‐allergy formula prescription

Low‐allergy formula was usually initiated by GPs, for a documented indication of CMA, or less commonly, intolerance (Table S7). EHF was usual as a first prescription (69% CMA overdiagnosis, 50% confirmed CMA), but similar numbers used an alternative EHF or AAF where a second prescription was provided. Low‐allergy formula was prescribed for median 10 months (1, 16) and 272 litres (26, 448) in CMA overdiagnosis or 9 months (3, 22) and 182 litres (28, 389) in confirmed CMA (Table S8). Total cost was a median of £1214 (104, 2649) for CMA overdiagnosis versus £854 (164, 1908) for confirmed CMA. We compared patterns of low‐allergy formula prescription in BEEP with national data for England in 2015 (Tables S9–S12). These show that documented prescribing in BEEP occurred at a similar level (15.5 litres/birth) to England data (14.5 litres/birth) or to prescriptions in the BEEP primary care practices in the previous year (13.6 litres/birth). However, there was increased prescription of AAF in BEEP 8.8 litres/birth, compared with 4.9 litres in England and 4.8 litres in the BEEP primary care practices—and less prescribing of soya formula. Assuming similar rates of prescribing for participants with missing data as in those with available primary care records, the prescribing rate for EHF was 33% higher in the BEEP cohort than in England (11.7 litres/birth vs. 8.8 litres) and for AAF >3‐fold higher in BEEP than in England (15.4 litres/birth vs. 4.9 litres). For all participants with repeated low‐allergy formula prescriptions, the estimated volume consumed per day was a median of 1.01 litres (0.86, 1.20) for AAF compared with 0.64 litres (0.42, 0.89) for EHF (*p* = .001) (Tables S13 and S14).

In those prescribed low‐allergy formula, skin symptoms were more commonly recorded at the time of low‐allergy formula prescription in confirmed CMA (90%) compared with CMA overdiagnosis (42%). Prescription of other medications was common in this sub‐group, especially Gaviscon® infant (43% CMA overdiagnosis, 30% confirmed CMA).

### 3.5 Evaluation of participant‐level risk factors for CMA overdiagnosis

We evaluated potential participant‐level risk factors for CMA overdiagnosis (Table 2, Tables S15–S17). Participants with true CMA (*n* = 19) were excluded from these analyses. In multivariate analysis, maternal report of antenatal use of antibiotic prescription during pregnancy (included as a potential marker of healthcare‐seeking behaviour),^31^, ^32^, ^33^ was significantly associated with CMA overdiagnosis (parent‐reported OR 1.79, 95% CI 1.19–2.70, *p* < .006; primary care record OR 2.11, 95% CI 1.30–3.42, *p* < .003; low‐allergy formula prescription OR 2.36, 95% CI 1.33–4.18, *p* < .003). Exclusive formula feeding from birth was significantly associated with low‐allergy formula prescription (OR 2.50, 95% CI 1.31–4.75, *p* < .005) but not with other measures of CMA overdiagnosis. We explored the same participant‐level risk factors for low‐allergy formula prescription within the population who had a primary care record of milk hypersensitivity (*n* = 124) (Table S18). Maternal age (OR 0.85, 95% CI 0.74–0.97, *p* = .02) and age of the child at diagnosis (OR 0.97, 95% CI 0.94–1.00, *p* = .02) were associated with reduced odds of low‐allergy formula prescription.

**TABLE 2:** Participant characteristics and CMA overdiagnosis.

	Parent‐reported milk reaction				Primary care record of cow's milk hypersensitivity				Primary care record of low‐allergy formula prescription			
	Adjusted (*n* = 777)^a^		Adjusted (*n* = 777)^b^		Adjusted (*n* = 758)^a^		Adjusted (*n* = 758)^b^		Adjusted (*n* = 757)^a^		Adjusted (*n* = 757)^b^	
	OR (95% CI)	*p*‐Value	OR (95% CI)	*p*‐Value	OR (95% CI)	*p*‐Value	OR (95% CI)	*p*‐Value	OR (95% CI)	*p*‐Value	OR (95% CI)	*p*‐Value
Maternal age	0.99 (0.95–1.03)	.67	–	–	0.99 (0.94–1.04)	.68	–	–	0.96 (0.91–1.02)	.24	–	–
White maternal ethnicity	0.85 (0.45–1.63)	.64	–	–	0.61 (0.30–1.24)	.17	–	–	0.78 (0.31–1.97)	.60	–	–
Antibiotics used in pregnancy	1.70 (1.12–2.59)	.01	1.79 (1.19–2.70)	.006	2.00 (1.22–3.28)	.006	2.11 (1.30–3.42)	.003	2.25 (1.25–4.05)	.007	2.36 (1.33–4.18)	.003
More than one first‐degree relative with atopic disease	0.92 (0.59–1.43)	.71	–	–	0.89 (0.53–1.51)	.67	–	–	1.11 (0.59–2.09)	.76	–	–
No other children in household	0.91 (0.59–1.40)	.66	–	–	0.83 (0.49–1.40)	.48	–	–	1.10 (0.59–2.04)	.77	–	–
Maternal anxiety/depression on EQ‐5D (at baseline)	0.70 (0.41–1.20)	.19	–	–	0.81 (0.43–1.52)	.52	–	–	0.87 (0.41–1.84)	.72	–	–
Maternal EQ‐5D health state at baseline	0.99 (0.97–1.00)	.11	–	–	0.98 (0.97–1.00)	.09	–	–	0.99 (0.97–1.01)	.46	–	–
Exclusive formula feeding from birth to 6 months old	1.50 (0.89–2.52)	.13	–	–	1.79 (0.98–3.25)	.06	1.72 (0.96–3.09)	.07	2.51 (1.30–4.86)	.006	2.50 (1.31–4.75)	.005
Family decile of English Index of Multiple Deprivation 2015	1.00 (0.93–1.08)	.97	–	–	1.03 (0.94–1.13)	.56	–	–	1.02 (0.91–1.15)	.68	–	–

*Note*: Logistic regression analysis comparing participant characteristics with CMA overdiagnosis, excluding the 19 confirmed milk‐allergic participants. CMA overdiagnosis was defined by parent‐reported milk reaction (*n* = 195), positive mention of reaction to milk in the primary care records (*n* = 124) and prescription of low‐allergy formula in the records (*n* = 77). Participants who did not answer any of the screening questions (*n* = 166), the primary care records that were not received (*n* = 40), and the participants with a positive mention of a reaction to milk whose prescription records were missing (*n* = 5) were excluded from analysis. Remaining BEEP study participants who did not answer yes to any of the screening questions and participants who did not have a mention of a milk reaction in the primary care records or prescription of low allergy formula were considered not to have CMA overdiagnosis. Values were adjusted to consider the association of all participant variables together on each outcome. Odds ratio (OR) >1 shows a positive association between each variable and the outcome. Confidence intervals are 95% and *p*‐value <.05 indicates statistical significance.

^a^ Step 1 of backward stepwise regression including all co‐variates.

^b^ Final step of backward stepwise regression.

To investigate the potential impact of missing data on findings, we compared characteristics of participants with and without missing values in the risk factors (Tables S19–S21), undertook a sensitivity analysis excluding EQ5D variables, which had the highest rate of missingness (Tables S22–S24) and undertook multiple imputation (Tables S25–S27). Findings continued to support an association between maternal reports of antibiotic prescription during pregnancy and all CMA overdiagnosis outcomes; and between exclusive formula feeding from birth and low‐allergy formula prescription.

### 3.6 Evaluation of practice‐level risk factors for CMA overdiagnosis

We evaluated potential primary care practice‐level risk factors for CMA overdiagnosis (Table 3, Tables S28–S31). Practice low‐allergy formula prescribing rate (litres/infant aged <1 year) was significantly associated with CMA overdiagnosis (parent‐reported OR 1.03, 95% CI 1.02–1.05, *p* < .001; primary care record OR 1.04, 95% CI 1.02–1.06, *p* < .001; low‐allergy formula prescription OR 1.04, 95% CI 1.02–1.07, *p* < .001). Practice antibiotic prescribing rate (a marker of overprescribing^20^, ^21^, ^23^) was not positively associated with CMA overdiagnosis. Indeed, there was a weak inverse association between practice antibiotic prescribing and the three measures of CMA overdiagnosis; which remained when antibiotic prescribing was adjusted based on the demographic structure of the practice population (STAR‐PU). Other practice features such as prescribing rates for AAI and reflux treatments, deprivation and local guideline recommendations were not associated with CMA overdiagnosis. When practice‐level and participant‐level variables were combined (Table 4, Tables S32–S34), associations were similar, including when multiple imputation was used to account for missing data (Tables S35–S37). These analyses found practice low‐allergy formula prescribing rates in the previous year and maternal reports of antibiotic prescription during pregnancy were associated with all three measures of CMA overdiagnosis; and exclusive formula feeding from birth with low‐allergy formula prescription.

**TABLE 3:** Practice‐level variables comparing participants with and without CMA overdiagnosis.

	Parent‐reported milk reaction				Primary care record of cow's milk hypersensitivity				Primary care record of low‐allergy formula prescription			
	Adjusted (*n* = 1193)^a^		Adjusted (*n* = 1193)^b^		Adjusted (*n* = 1153)^a^		Adjusted (*n* = 1153)^b^		Adjusted (*n* = 1148)^a^		Adjusted (*n* = 1148)^b^	
	OR (95% CI)	*p*‐Value	OR (95% CI)	*p*‐Value	OR (95% CI)	*p*‐Value	OR (95% CI)	*p*‐Value	OR (95% CI)	*p*‐Value	OR (95% CI)	*p*‐Value
Practice volume of low‐allergy formula prescription (litres per infant aged 0–1 year practice population)	1.03 (1.01–1.05)	.002	1.03 (1.02–1.05)	<.001	1.04 (1,02–1.06)	<.001	1.04 (1.02–1.06)	<.001	1.04 (1.02–1.07)	.001	1.04 (1.02–1.07)	<.001
Practice antibiotic prescriptions (Items per 1000 total practice population)	0.995 (0.99–1.00)	.05	0.996 (0.992–1.00)	.09	0.998 (0.997–1.00)	.02	0.999 (0.997–1.00)	.03	0.998 (0.996–1.00)	.02	0.998 (0.997–1.00)	.03
Practice decile of English Index of Multiple Deprivation (IMD) 2019	0.97 (0.91–1.03)	.33	–	–	0.96 (0.89–1.04)	.3	–	–	0.96 (0.88–1.05)	.36	–	–
Practice anti‐reflux prescriptions (Items per infant aged 0–1 year practice population)	1.03 (1.00–1.05)	.06	–	–	1.03 (1.00–1.06)	.09	–	–	1.02 (0.99–1.06)	.23	–	–
Practice Gaviscon® Infant prescriptions (Quantity per infant aged 0–1 year practice population)	1.00 (0.99–1.01)	.85	–	–	1.00 (0.99–1.01)	.60	–	–	1.00 (0.99–1.01)	.98	–	–
Practice AAI prescriptions (Items per 1000 aged 0–5 years practice population)	1.00 (1.00–1.01)	.67	–	–	1.00 (1.00–1.01)	.64	–	–	1.00 (0.99–1.01)	.69	–	–
CCG CMA guidelines highlight reproducibility or specificity as criteria for CMA diagnosis	1.59 (0.96–2.63)	.07	–	–	1.57 (0.86–2.86)	.14	–	–	1.64 (0.81–3.32)	.17	–	–
Antibiotic Items/STARPU (Average of 4 quarters for 2014)^c^	0.97 (0.01–1.58)	.10	0.10 (0.01–1.30)	.08	0.16 (0.00–0.52)	.02	0.03 (0.00–0.72)	.03	0.01 (0.00–0.49)	.02	0.02 (0.00–0.73)	.03

*Note*: Logistic regression comparing practice‐level variables of those participants with CMA overdiagnosis with the remaining BEEP study cohort without CMA overdiagnosis, excluding the 19 confirmed milk‐allergic participants. CMA overdiagnosis was defined by parent‐reported milk reaction (*n* = 195), positive mention of reaction to milk in the primary care records (*n* = 124) and prescription of low‐allergy formula in the records (*n* = 77). Participants who did not answer any of the screening questions (*n* = 166), the primary care records that were not received (*n* = 40), and the participants with a positive mention of a reaction to milk whose prescription records were missing (*n* = 5) were excluded from the analysis. Remaining BEEP study participants who did not answer yes to any of the screening question and participants who did not have a mention of a milk reaction in the primary care records or prescription of low allergy formula were considered not to have CMA overdiagnosis. No data was available for 15 GP practices. Odds ratio (OR) >1 shows a positive association between each variable and each outcome. Confidence intervals are 95% and *p*‐value <.05 indicates statistical significance. Adjusted values consider the association of all variables together on the outcome.

^a^ Step 1 of backward stepwise regression including all co‐variates.

^b^ Final step of backward stepwise regression. Practice‐level variables were assessed for the year prior to the BEEP study (2014) except IMD which was recorded for 2019 as this was the year used in the BEEP clinical trial for participant IMD.

^c^ Antibiotic Items/STARPU was substituted for practice antibiotic prescription items as a predictor variable to adjust for gender and sex effects on antibiotic prescribing in the practice.

**TABLE 4:** Combined participant and practice‐level variables comparing participants with and without parent‐reported cow's milk hypersensitivity.

	Parent‐reported cow's milk hypersensitivity				Primary care record of cow's milk hypersensitivity				Primary care record of low‐allergy formula prescription			
	Adjusted (*n* = 529)^a^		Adjusted (*n* = 529)^b^		Adjusted (*n* = 515)^a^		Adjusted (*n* = 515)^b^		Adjusted (*n* = 514)^a^		Adjusted (*n* = 514)^b^	
	OR (95% CI)	*p*‐Value	OR (95% CI)	*p*‐Value	OR (95% CI)	*p*‐Value	OR (95% CI)	*p*‐Value	OR (95% CI)	*p*‐Value	OR (95% CI)	*p*‐Value
Maternal age	0.96 (0.91–1.01)	.12	–	–	0.97 (0.91–1.03)	.31	–	–	0.95 (0.88–1.02)	.15	–	–
White maternal ethnicity	0.56 (0.23–1.35)	.20	–	–	0.36 (0.14–0.95)	.04	0.43 (0.17–1.06)	.07	0.41 (0.13–1.22)	.11	–	–
Antibiotics used in pregnancy	1.95 (1.17–3.27)	.01	1.97 (1.19–3.26)	.008	2.09 (1.12–3.88)	.02	2.17 (1.19–3.97)	.01	2.10 (1.02–4.29)	.04	2.42 (1.22–4.82)	.01
More than one first degree relative with atopic disease	1.11 (0.64–1.92)	.72	–	–	1.33 (0.67–2.64)	.42	–	–	1.50 (0.67–3.34)	.32	–	–
No other children in household	1.00 (0.58–1.71)	.99	–	–	0.92 (0.48–1.78)	.80	–	–	1.15 (0.54–2.45)	.71	–	–
Maternal anxiety/depression on EQ‐5D (at baseline)	0.64 (0.32–1.29)	.21	–	–	0.91 (0.41–2.02)	.81	–	–	0.74 (0.28–1.96)	.54	–	–
Maternal EQ‐5D health state at baseline	0.98 (0.96–1.00)	.03	0.98 (0.96–1.00)	.06	0.98 (0.96–1.01)	.10	0.98 (0.96–1.00)	.07	0.98 (0.95–1.01)	.12	–	–
Exclusive formula feeding from birth to 6 months old	1.80 (0.96–3.37)	.07	1.73 (0.94–3.18)	.08	2.52 (1.23–5.19)	.01	2.39 (1.19–4.78)	.01	3.16 (1.42–7.02)	.005	2.87 (1.35–6.10)	.006
Practice volume of low‐allergy formula prescription (litres per infant aged 0–1 year practice population)	1.03 (0.96–1.05)	.11	1.03 (1.00–1.05)	.05	1.03 (1.00–1.06)	.08	1.04 (1.01–1.07)	.007	1.04 (1.02–1.07)	.001	1.04 (1.02–1.07)	<.001
Practice antibiotic prescriptions (Items per 1000 total practice population)	1.00 (0.99–1.00)	.78	–	–	0.99 (0.99–1.00)	.59	–	–	0.998 (0.996–1.00)	.02	–	–
Practice decile of English Index of Multiple Deprivation (IMD) 2019	1.04 (0.95–1.15)	.41	–	–	1.06 (0.94–1.19)	.38	–	–	0.96 (0.88–1.05)	.36	–	–
Practice anti‐reflux prescriptions (Items per infant aged 0–1 year practice population)	1.00 (0.95–1.05)	.99	–	–	1.00 (0.94–1.07)	.94	–	–	1.02 (0.99–1.06)	.23	–	–
Practice Gaviscon® Infant prescriptions (Quantity per infant aged 0–1 year practice population)	1.00 (0.99–1.01)	.81	–	–	1.00 (0.99–1.02)	.63	–	–	1.00 (0.99–1.01)	.98	–	–
Practice AAI prescriptions (Items per 1000 aged 0–5 years practice population)	1.00 (0.99–1.01)	.94	–	–	1.01 (1.00–1.01)	.32	–	–	1.00 (0.99–1.01)	.69	–	–
CCG CMA guidelines highlights reproducibility or specificity as criteria for CMA diagnosis	1.91 (1.01–3.63)	.05	1.86 (0.99–3.46)	.05	1.60 (0.73–3.53)	.24	–	–	1.64 (0.81–3.32)	.17	–	–

*Note*: Logistic regression comparing combined participant and practice‐level variables of those participants with CMA overdiagnosis with the remaining BEEP study cohort without CMA overdiagnosis, excluding the 19 confirmed milk‐allergic participants. CMA overdiagnosis was defined by parent‐reported milk reaction (*n* = 195), positive mention of reaction to milk in the primary care records (*n* = 124) and prescription of low‐allergy formula in the records (*n* = 77). Participants who did not answer any of the screening questions (*n* = 166), the primary care records that were not received (*n* = 40), and the participants with a positive mention of a reaction to milk whose prescription records were missing (*n* = 5) were excluded from analysis. Remaining BEEP study participants who did not answer yes to any of the screening questions and participants who did not have a mention of a milk reaction in the primary care records or prescription of low allergy formula were considered not to have CMA overdiagnosis. No data was available for 15 GP practices. Odds ratio (OR) >1 shows a positive association between each variable and each outcome. Confidence intervals are 95% and *p*‐value <.05 indicates statistical significance. Adjusted values consider the association of all variables together on the outcome.

^a^ Step 1 of backward stepwise regression including all co‐variates.

^b^ Final step of backward stepwise regression. Practice‐level variables were assessed for the year prior to the BEEP study (2014) except IMD which was recorded for 2019 as this was the year used in the BEEP clinical trial for participant IMD.

## 4 DISCUSSION

### 4.1 Main findings

In this analysis of a clinical trial birth cohort with prospective assessment of CMA diagnosis, we found that in those participants who did not have confirmed CMA, about 16% of parents reported a reaction to milk in their child by age 2 years, 11% had primary care records documenting a milk hypersensitivity and 9% of children were prescribed a low‐allergy formula during the first 2 years of life. In those without confirmed CMA, we identified primary care practitioners as initiating overdiagnosis and low allergy formula prescriptions most commonly. Gastrointestinal symptoms were the most common concern triggering CMA overdiagnosis and unnecessary prescription. CMA overdiagnosis presented at a median of 49 days old and was diagnosed in median of 37 days from symptom onset. Low‐allergy formula exposure occurred for a median of 10 months, at a median estimated consumption of 272 litres. Daily low‐allergy formula consumption appeared to be greater for AAF (median 1 litre per day) than EHF (median 0.64 litres per day). We identified risk factors for CMA overdiagnosis as maternal report of antibiotic prescription during pregnancy and higher practice‐based prescribing of low‐allergy formula. Exclusive formula feeding from birth was associated with increased risk of low‐allergy formula use but was not consistently associated with other markers of CMA overdiagnosis—indeed, CMA overdiagnosis commonly occurred in breastfed infants. Our findings suggest that the prescribing habits of primary care practitioners for low‐allergy formula may be important for CMA overdiagnosis. Maternal use of antibiotics in pregnancy was included in these analyses as a potential marker for increased healthcare‐seeking behaviour,^31^, ^32^, ^33^ and further work is needed to identify whether the healthcare‐seeking behaviour of some mother/infant dyads puts them at increased risk for CMA overdiagnosis.

The high rates of CMA overdiagnosis in BEEP are consistent with other studies suggesting that CMA is over‐reported by parents, perhaps more so than other food allergies, and many low‐allergy formula prescriptions are for children without CMA.^2^, ^34^, ^35^, ^36^ The findings build on recent work which estimated 2.2% of children were prescribed low‐allergy formula for CMA in Norway in the same time period, and 4.9% of United States store purchases of formula were low‐allergy formula for CMA in 2017.^2^, ^3^ These figures rose to 6.9% and 7.6% by 2020 and 2019 respectively, closer to our estimate of 8.7%.^2^, ^3^ Based on population prevalence of CMA and formula feeding rates in the local population, these data suggest that over 90% of low‐allergy formula prescription is outside of the context of a reproducible CMA diagnosis.^2^, ^3^ Our findings suggest that low‐allergy formula is being used for managing gastrointestinal symptoms, especially diarrhoea, vomiting and reflux and that multiple healthcare practitioners and parents are all contributing to this process. Maternal dietary restrictions are commonly undertaken and advised. This is something which is commonly advised in milk allergy guidelines but is not evidence‐based and may be harmful.^11^, ^28^, ^35^, ^36^, ^37^, ^38^, ^39^ The increased daily volume of AAF consumption compared with EHF may reflect a safety issue related to a failure AAF to induce normal satiety mechanisms, and requires further confirmation.^40^ Ultraprocessed foods are thought to promote obesity due to inadequate induction of satiety.^41^ AAF, which is glucose syrup based and contains no peptides, may have a similar effect. Previous work has suggested there is a dose–response relationship between glucose‐syrup‐based formula consumption during infancy and increased early childhood obesity^3^, ^6^, ^7^, ^8^, ^9^ Finally, it is possible that CMA overdiagnosis in breastfed infants may actually promote the development of IgE‐mediated CMA through the delayed introduction of cow's milk antigen to the infant diet.^42^ However, evidence for early cow's milk introduction and CMA prevention is currently inconclusive.^43^ Our findings have implications for strategies such as prescribing restrictions, to prevent CMA overdiagnosis and excessive prescribing of specialised low‐allergy formula products. One important target for interventions could be primary care practitioners caring for families who are concerned about gastrointestinal symptoms in young infants.

### 4.2 Study strengths and limitations

The main strength of this study is the unique dataset it presents, where confirmed CMA and over‐reporting of CMA by parents were well‐characterised. Limitations of these findings include the specific clinical trial population studied, where families had a history of atopic disease. Clinical trial populations tend to differ from the general population, and for BEEP we found higher rates of white maternal ethnicity and higher socioeconomic status than the general population of England (Table S38). Prescription rates for low‐allergy formula were also estimated to be higher than the general population, especially for AAF, and the reasons for this are not completely clear. Healthcare‐seeking behaviour, especially in relation to allergy issues, may be different in the BEEP cohort from the general population. Antenatal prescription of antibiotics was not verified from prescription records and relied on maternal recollection which may affect the validity of this risk factor for CMA overdiagnosis. Primary care records did not consistently document who raised an initial concern about milk hypersensitivity. Therefore, in some cases where we judged the primary care physician as being most likely to have raised the initial concern, this had not been explicitly stated in the record. This may be an important limitation when considering targets for intervention, although it is also relevant that records only documented primary care practitioners refuting milk hypersensitivity in two cases. Missing outcome data for some participants means that estimates for CMA overdiagnosis rates in the BEEP population are approximate. The process for identifying confirmed CMA is unlikely to have identified all cases of non‐IgE mediated CMA. For example, cow's milk food protein‐induced enterocolitis syndrome (FPIES) is estimated to affect 0.34% of infants yielding an expected incidence of four to five cases within the BEEP cohort.^44^ Robust elimination and re‐introduction procedures were not well documented in primary care records and were not undertaken routinely in the BEEP trial for all participants with concerns about CMA and negative skin prick tests. The health impact on mother and child, and the impact on public health systems resources, of the labelling by parents and healthcare practitioners of possible milk hypersensitivity needs further exploration.

In conclusion, CMA overdiagnosis affects 11%–16% of young children and is mainly triggered by gastrointestinal symptoms which start in the first weeks of life. Half of infants with parent‐reported milk reactions are prescribed a low‐allergy formula, for a median duration of 10 months. CMA overdiagnosis carries a significant financial burden, can adversely affect breastfeeding and carries long‐term health risks for children associated with exposure to high levels of free sugars in low‐allergy formula.

## AUTHOR CONTRIBUTIONS

Dr Boyle had full access to all of the data in the study and took responsibility for the integrity of the data and the accuracy of the data analysis. Concept and design: Boyle, Allen, Bradshaw, Moriarty. Acquisition, analysis or interpretation of data: Allen, Jackson, Milkova, Wing, Li, Bradshaw, Moriarty, Boyle. Drafting of the manuscript: Allen, Boyle. Critical revision of the manuscript for important intellectual content: All authors. Statistical analysis: Allen, Jackson, Milkova, Wing, Li, Bradshaw, Moriarty, Boyle. Supervision: Boyle, Bradshaw, Moriarty.

## CONFLICT OF INTEREST STATEMENT

RJB declares payment for expert witness work in legal cases related to food anaphylaxis and an infant formula health claim, and payment for editorial work from Cochrane, Wiley and the British Society for Allergy and Clinical Immunology. HCW directed the NIHR Health Technology Assessment Programme 2015 to 2020 which funded the BEEP study. He played no part in the decision to fund the study. All other authors declare no conflict of interest.

## Supporting information


Appendix S1

## Data Availability

The data that support the findings of this study are available on request from the corresponding author. The data are not publicly available due to privacy or ethical restrictions.
