# Supplementary material for: Prevalence and risk factors for milk allergy overdiagnosis in the BEEP trial cohort
Source: Allergy. 2024 Jun 20;80(1):148–60. doi: 10.1111/all.16203 (PMC11724250; doi:10.1111/all.16203)
Supplement: Supplementary file 1 — Appendix S1 [file ALL-80-148-s001.docx]

**Supplementary Material**

**Prevalence and risk factors for milk allergy overdiagnosis in the BEEP trial cohort**

Hilary I Allen^1^, *Olivia Wing^1^, *Dara Milkova^1^, *Emilia Jackson^2^, *Karen Li^1^, Lucy E Bradshaw^3^, Laura Wyatt^3^, Rachel Haines^3^, Miriam Santer^4^, Andrew W Murphy^5^, Sara J Brown^6^, Maeve Kelleher^7^, Michael Perkin^8^, Nicola Jay^9^, Timothy D H Smith^10^, Frank Moriarty^11^, Alan A Montgomery^3^, Hywel C Williams^2^, Robert J Boyle^1^ *These authors made equal contributions

**Author Institutions:** *^1^National Heart and Lung Institute, Imperial College London, London, United Kingdom.*

*^2^Centre of Evidence Based Dermatology, University of Nottingham, Nottingham, United Kingdom*

*^3^Nottingham Clinical Trials Unit, School of Medicine, University of Nottingham, Nottingham, United Kingdom*

*^4^Primary Care Research Centre, University of Southampton, Southampton, United Kingdom*

*^5^ Department of General Practice & HRB Clinical Trial Network Primary Care Ireland, University of Galway, Ireland.*

*^6^Centre for Genomic and Experimental Medicine, University of Edinburgh, Edinburgh, Scotland*

*^7^Children’s Health Ireland, Crumlin Children’s Hospital, Dublin. Ireland*

*^8^Population Health Research Institute, St George's University of London, London, United Kingdom*

*^9^Sheffield Children’s NHS Foundation Trust, Sheffield, United Kingdom*

*^10^NIHR Clinical Research Network North West Coast, Liverpool, United Kingdom*

*^11^School of Pharmacy and Biomolecular Sciences (PBS), Royal College of Surgeons, Dublin, Ireland.*

Table of Contents

[Supplementary Methods 5](#_Toc166338115)

[Data extraction and classification 5](#_Toc166338116)

[Sensitivity analyses for logistic regression 7](#_Toc166338117)

[Clustering 7](#_Toc166338118)

[Multiple imputation 7](#_Toc166338119)

[Table S1. Primary care practice prescription data analysis 9](#_Toc166338120)

[Table S2. Primary Care Practice CMA guideline analysis 11](#_Toc166338121)

[Table S3. Clinical details for participants with confirmed CMA 12](#_Toc166338122)

[Table S4. Performance of screening questions in relation to confirmed primary care medical record of cow’s milk hypersensitivity 14](#_Toc166338123)

[Table S5. Diagnostic process. 15](#_Toc166338124)

[Table S6: Diet and Feeding 17](#_Toc166338125)

[Table S7: Low-allergy formula prescribed during the first year of life 19](#_Toc166338126)

[Table S8: Volume and cost of low-allergy formula prescribed to BEEP trial participants 21](#_Toc166338127)

[Table S9: Estimations for incidence of cow’s milk hypersensitivity and low-allergy formula prescription in BEEP study cohort 23](#_Toc166338128)

[Table S10: Method for calculating estimated volume and cost of low-allergy formula per infant born 24](#_Toc166338129)

[Table S11: Documented and estimated volume and cost of low-allergy formula in BEEP study cohort 25](#_Toc166338130)

[Table S12: Volume and cost of low-allergy formula per infant born 27](#_Toc166338131)

[Table S13: Prescription of extensively hydrolysed formula and amino acid formula in BEEP cohort 29](#_Toc166338132)

[Table S14: Repeat prescriptions of extensively hydrolysed formula and amino acid formula in BEEP cohort categorising participants according to the main formula prescribed 31](#_Toc166338133)

[Table S15: Participant characteristics and parent-reported cow’s milk reaction 32](#_Toc166338134)

[Table S18: Participant characteristics and primary care record of a low-allergy formula prescription in participants with a primary care record of cow’s milk hypersensitivity 38](#_Toc166338135)

[Table S19. Missingness of participant-level predictors and CMA outcome variables in the BEEP trial dataset 40](#_Toc166338136)

[Table S20. Characteristics of BEEP study participants without confirmed CMA, comparing complete versus missing data for predictor variables with high levels of missingness. 41](#_Toc166338137)

[Table S21. Characteristics of BEEP participants with confirmed CMA, comparing complete versus missing data for predictor variables with high levels of missingness. 43](#_Toc166338138)

[Table S22: Participant characteristics and parent-reported cow’s milk reaction (excluding EQ5D) 45](#_Toc166338139)

[Table S23. Participant characteristics and primary care record of cow’s milk hypersensitivity (excluding EQ5D) 46](#_Toc166338140)

[Table S24. Participant characteristics and primary care record of low-allergy formula prescription (excluding EQ5D) 47](#_Toc166338141)

[Table S25: Participant characteristics and parent-reported cow’s milk reaction (multiple imputation, 100 imputations). 48](#_Toc166338142)

[Table S26: Participant characteristics and primary care record of cow’s milk hypersensitivity (multiple imputation, 100 imputations). 49](#_Toc166338143)

[Table S27: Participant characteristics and primary care record of low-allergy formula prescription (multiple imputation, 100 imputations). 51](#_Toc166338144)

[Table S28. Clustering of BEEP participants in primary care practices 53](#_Toc166338145)

[Table S29: Practice-level variables comparing participants with and without parent-reported cow’s milk hypersensitivity 54](#_Toc166338146)

[Table S30: Practice-level variables comparing participants with and without a primary care record of cow’s milk hypersensitivity 56](#_Toc166338147)

[Table S31: Practice-level variables comparing participants with and without a primary care record of low-allergy formula prescription 58](#_Toc166338148)

[Table S32: Combined participant and practice-level variables comparing participants with and without parent-reported cow’s milk hypersensitivity 60](#_Toc166338149)

[Table S33: Combined participant and practice-level variables comparing participants with and without a primary care record of cow’s milk hypersensitivity 62](#_Toc166338150)

[Table S34: Combined participant and practice-level variables comparing participants with and without a primary care record of low-allergy formula prescription 64](#_Toc166338151)

[Table S35: Combined participant and practice-level variables comparing participants with and without parent-reported cow’s milk reaction (multiple imputation, 100 imputations) 66](#_Toc166338152)

[Table S36: Combined participant and practice-level variables comparing participants with and without a primary care record of cow’s milk hypersensitivity (multiple imputation, 100 imputations) 68](#_Toc166338153)

[Table S37: Combined participant and practice-level variables comparing participants with and without a primary care record of low-allergy formula prescription (multiple imputation, 100 imputations) 70](#_Toc166338154)

[Table S38: BEEP participant characteristics compared to general population England 2014 72](#_Toc166338155)

[Figure S1. Overlap between categories of CMA overdiagnosis 73](#_Toc166338156)

[Figure S2. Timing of initial symptoms and diagnosis of possible cow’s milk hypersensitivity 74](#_Toc166338157)

[Figure S3. Timing of dietary interventions for possible cow’s milk hypersensitivity 76](#_Toc166338158)

[Figure S4. Timing of low-allergy formula prescription for possible cow’s milk hypersensitivity 77](#_Toc166338159)

[Figure S5. Timing of extensively hydrolysed and amino acid formula prescription for possible cow’s milk hypersensitivity 79](#_Toc166338160)

[Figure S6. Symptoms recorded at time of first reaction to milk in children with confirmed CMA 81](#_Toc166338161)

[References 82](#_Toc166338163)

# Supplementary Methods

Letters to participating families and primary care practitioners and a copy of the ethics approval letter are available in an online repository (<https://osf.io/y7mkr/>), together with the R code used for extracting population-based prescribing data from the National Health Service Business Services Authority website.

## Data extraction and classification

Primary outcome data collected was primary care record of milk reaction and prescription or documented use of a low-allergy low allergy formula. Other data collected included the most definitive diagnosis given by a healthcare professional, age of child at initial symptoms and diagnosis, nature of initial symptoms and diagnosis, role of person who raised initial concern of a reaction to milk, role of healthcare professionals who diagnosed milk allergy and who prescribed low-allergy formula, indication for low-allergy formula prescription, infant feeding status at diagnosis and at prescription of low-allergy formula, documentation of advice for maternal or infant dietary restriction, presence or absence of dietician review, type(s) of low-allergy formula, age at initial and final prescription, duration, volume and cost of low-allergy formula, other prescribed medicines, and primary care record of any formal diagnostic assessment for CMA. Role of person who raised initial concern of a reaction to milk was interpreted as the GP for those cases where the GP clearly documented that they were concerned about a reaction to milk and was assumed as the GP in those cases where no other healthcare professional/parent was clearly identified and the initial consultation was with the GP.

Confirmed CMA diagnosis was defined as participants confirmed milk-allergic through formal diagnostic assessment (skin prick testing, oral food challenge and expert panel review) undertaken in the original BEEP study at 2 years old and participants who met BEEP criteria but were beyond the age 2 years old window for diagnosis in the original study. BEEP criteria for diagnosis of CMA were allergic reaction to cow’s milk on supervised oral food challenge at age 2 years or expert panel consensus diagnosis, based on available information from clinical history and skin prick testing undertaken as part of BEEP, and additional information collected from primary care and/or secondary care records and/or supplementary telephone interview with participating families. Expert panel consensus followed published guidance. (1,2) In BEEP, IgE-mediated CMA at age 2 years was diagnosed in 17 participants (1 by oral food challenge and 16 by expert panel consensus). For this study of CMA overdiagnosis, a further 2 participants were identified who had CMA, but didn’t have IgE-mediated CMA at the age of 2 years. One participant had non-IgE CMA proven by oral food challenge, and one participant had IgE-mediated CMA which resolved before age 2 years.

CMA overdiagnosis was defined in three ways. For all three evaluations, participants with proven IgE-mediated or non-IgE mediated CMA through BEEP trial assessments were excluded from analysis. The first definition was ‘parent-reported milk reaction’, categorised as present for participants whose parent/carer answered yes to at least one of the 3 milk allergy screening questions used at 1 and 2 years in the original BEEP clinical trial, shown in Table 1. The second definition was ‘primary care record of milk hypersensitivity’, categorised as present for participants whose primary care record included description of a reaction or possible reaction attributed to cow’s milk ingestion. The third definition was ‘low-allergy formula prescription’, categorised as present for participants whose primary care record indicated that a soya, extensively hydrolysed or amino-acid formula had been prescribed.

## Sensitivity analyses for logistic regression

Sensitivity analyses were performed for logistic regression analysis of associations between participant features and CMA overdiagnosis. Due to a relatively large amount of missing data for EQ5D measurements in the BEEP trial dataset (Tables S19-S21), we repeated multivariate logistic regression analyses excluding EQ5D (Tables S22-S24). We also undertook multiple imputation analysis for variables with significant missing data (Tables S25-S27). For logistic regression analysis of associations between practice characteristics and CMA overdiagnosis, we undertook a sensitivity analysis where antibiotic items per STAR-PU was substituted in place of total antibiotic items per 1000 practice population, in order to understand whether practice population demographic structure was likely to influence relationships seen between antibiotic prescription rates and CMA overdiagnosis (Tables S28-31).

## Clustering

The mixed models function within SPSS was used to adjust for clustering within practices when assessing associations between practice-level characteristics and CMA overdiagnosis. This method employed generalised linear mixed models using GP practice code to identify clusters (Tables S28-S31).

## Multiple imputation

Multiple imputation details are reported following previous published guidance (3) Data were missing in the BEEP cohort for the predictor and outcome variables of interest shown in Table S19. Tables S20 (CMA overdiagnosis) and Table S21 (confirmed CMA) outline the number of complete and missing values for each predictor and outcome variable. Participants with missing predictor variables were broadly similar to participants with complete predictor variables in terms of the predictors and outcomes. Although complete case analysis excluding the missing values can be undertaken, it reduces statistical power, and the risk of bias is reported with missing values approaching 30%. (4) Multiple imputation is suggested as a method of managing missing data (3,5). Multiple imputation was performed using the automatic multiple imputation function in SPSS. The imputation method was fully conditional specification, Markov chain Monte Carlo (MCMC) which is suitable for data with a random pattern of missing values. The following predictor variables were imputed into the model: EQ5D health state at baseline (answered antenatally or shortly after birth), EQ5D anxiety at baseline (answered antenatally or shortly after birth), Infant feeding status from birth to 6 months, Decile of English Index of Multiple Deprivation 2015, Maternal use of antibiotics in pregnancy. The following outcome variables were imputed into the model: Parent reported reaction to milk, Primary care record of milk reaction, Primary care record of low-allergy formula prescription. It has been suggested that a high number of imputations should be performed to improve statistical power. (6) One hundred datasets were imputed for both predictor and outcome variables, to reduce possible attenuation of the association by only imputing for missing predictor variables (Table S25-S27). (3,6) The percentage of participants in the imputed and complete dataset were similar for each of the 3 CMA overdiagnosis outcomes (Table S20). Logistic regression analysis was performed on the imputed dataset in the same manner as the complete dataset (Table S25-S27). Multiple imputation was used for the missing participant-level characteristics only as missing data for practice-level characteristics was minimal (n=19/1375) (Tables S29-S31).

#

# Table S1. Primary care practice prescription data analysis

| **Name** | **Type / strength** | **BNF Code** |
| --- | --- | --- |
| Aptamil pepti 1 | ehf | 090402000BBNZA0 |
| Aptamil pepti 2 | ehf | 090402000BBTQA0 |
| Farleys soya | soya | 090401000BBFJA1 |
| Infasoy | soya | 090401000BBFLA6 |
| Neocate LCP | aaf | 090401000BBNQA0 |
| Neocate | aaf | 090401000BBBSA0 |
| Nutramigen | ehf | 090401000BBDIAZ |
| Nutramigen Puramino | aaf | 090401000BBNMA0 |
| Nutramigen 1 Lipil | ehf | 090401000BBLUA0 |
| Nutramigen 2 Lipil | ehf | 090401000BBLPA0 |
| Similac Alimentum | ehf | 090900000BBKRA0 |
| SMA Alfamino | aaf | 090900000BBKWA0 |
| SMA Althera | ehf | 090900000BBKQA0 |
| SMA Wysoy | soya | 090401000BBGZBD |
| Epipen Jr Auto-injector | 0.15mg | 0304030C0BEAAA2 |
| Adrenaline | 150 mcg 0.3ml | 0304030C0AAA2A2 |
| Anapen Junior | 150 mcg 0.3ml | 0304030C0BGABA2 |
| Jext | 150 mcg 0.15ml | 0304030C0BHAABF |
| Emerade | 150 mcg 0.15ml | 0304030C0BIAABF |
| Domperidone | 1.9mg/5ml oral suspension | 0406000J0AAALAL |
| Domperidone 1mg/ml oral suspension sugar free | 1mg/ml oral suspension sugar free | 0406000J0AAACAC |
| Gaviscon® infant oral powder sachets |  | 0101021B0BEADAJ |
| Instant Carobel powder |  | 0913161B0BBAAAF |
| Lansoprazole | 15mg orodispersible tablets | 0103050L0AAAGAG |
| Losec MUPS | 10mg gastro-resistant tablets | 0103050P0BBADAM |
| Omeprazole | 10mg dispersible gastro-resistant tablets | 0103050P0AAAMAM |
| Omeprazole | 10mg gastro-resistant capsules | 0103050P0AAAFAF |
| Omeprazole | 2.5mg/5ml oral suspension | 0103050P0AAALAL |
| Omeprazole | 2mg/5ml oral liquid | 0103050P0AABABA |
| Omeprazole | 3.5mg/5ml oral liquid | 0103050P0AABRBR |
| Omeprazole | 3mg/5ml oral liquid | 0103050P0AABFBF |
| Omeprazole | 4mg/5ml oral liquid | 0103050P0AAASAS |
| Omeprazole | 5mg/5ml oral solution | 0103050P0AABSBS |
| Omeprazole | 5mg/5ml oral suspension | 0103050P0AABNBN |
| Ranitidine | 1.5mg/5ml oral liquid | 0103010T0AAAZAZ |
| Ranitidine | 3mg/5ml oral liquid | 0103010T0AABHBH |
| Ranitidine | 4mg/5ml oral liquid | 0103010T0AAARAR |
| Ranitidine | 5mg/5ml oral liquid | 0103010T0AABQBQ |
| Ranitidine | 5mg/5ml oral suspension | 0103010T0AABRBR |
| Ranitidine | 75mg/5ml oral solution sugar free | 0103010T0AAAEAE |

BNF, British National Formulary. The list includes all prescription items evaluated in analysis of practice-level risk factors for CMA overdiagnosis

#

# Table S2. Primary Care Practice CMA guideline analysis

| Criteria | Number of CCGs |
| --- | --- |
| No Guidelines | 20 |
| Partial guidelines | 10 |
| Complete guidelines | 50 |
| Differentiated between IgE and non-IgE mediated symptoms | 40 |
| Highlighted the need for reproducibility of symptoms | 9 |
| Advice on excluding differential diagnoses | 3 |
| Not tolerating other forms of cow’s milk mentioned | 10 |
| Faltering growth mentioned as key symptom | 39 |
| States that generally breast milk is the optimal milk for infants with or without CMA | 39 |
| Recommends giving limited supply of low-allergy formula, until full diagnosis | 23 |
| Recommends re-introduction of cow's milk after low-allergy formula feeding trial | 33 |
| Recommends re-introduction of cow's milk after maternal dietary exclusion trial | 30 |
| Includes recommendations on review and discontinuation of formula | 34 |
| Recommends formula to be stopped when over 12 months with a good, mixed diet and normal growth OR rechallenge | 21 |

Data summarise local Clinical Commissioning Group CMA guidelines for primary care practices which were evaluated for potential practice-level risk factors for CMA overdiagnosis. Criteria evaluated are based on a previous Delphi Consensus on detection and management of CMA in children under age 2 years (7) Data was categorised ‘yes’ if the CCG guideline referred to the criteria identified from the Delphi consensus study, ‘no’ if the criteria were not mentioned in the CCG guideline and ‘missing’ if there was no guideline. If the CCG CMA guideline referred to either reproducibility or specificity as being relevant to CMA diagnosis, this was coded as ‘yes’ and if neither were referred to this was coded as ‘no’ for the purposes of further analyses (See Tables S29-S37).

# Table S3. Clinical details for participants with confirmed CMA

| **Participant** | **Reaction(s) reported** | **Cow’s milk skin prick test result at aged 2** | **Consumption of cow’s milk at age 2 years without reaction** | **Diagnosis** |
| --- | --- | --- | --- | --- |
| 1 | Reaction at oral food challenge. Hives, lip angioedema and diarrhoea within 30 minutes of third dose. | 5mm | No | IgE CMA |
| 2 | More than two episodes of skin symptoms within 30 minutes of exposure to cow’s milk | 10mm | No | IgE CMA |
| 3 | Reaction to a biscuit containing cow’s milk. Hives, vomiting, cough and wheeze within 30 minutes | 20mm | No | IgE CMA |
| 4 | Delayed vomiting reaction following oral food challenge, consistent with previous delayed vomiting reactions to cow’s milk | 2mm | Tolerating baked milk | Non-IgE CMA |
| 5 | Repeated hives within 30 minutes of exposure to cow’s milk | 4mm | Tolerating baked milk | IgE CMA |
| 6 | Repeated skin rashes within 30 minutes of exposure | 9mm | No | IgE CMA |
| 7 | Vomiting, hives, drowsy within 30 minutes of consuming cow’s milk | 7mm | No | IgE CMA |
| 8 | Repeated episodes of hives with or without gastrointestinal symptoms within 30 minutes of exposure to cow’s milk, including after baked milk challenge | 7mm | No | IgE CMA |
| 9 | Repeated skin symptoms within 30 minutes of exposure to cow’s milk | 8mm | Tolerating baked milk | IgE CMA |
| 10 | Repeated episodes of hives and vomiting within 30 minutes of exposure to cow’s milk, including baked milk | 12mm | No | IgE CMA |
| 11 | Repeated episodes of hives within 30 minutes of exposure to cow’s milk | 10mm | Tolerating baked milk | IgE CMA |
| 12 | Repeated episodes of hives within 30 minutes of exposure to cow’s milk | 10mm | Tolerating baked milk | IgE CMA |
| 13 | Hives, angioedema, pruritus, diarrhoea and wheezing within an hour of exposure to cow’s milk | 7mm | No | IgE CMA |
| 14 | Repeated episodes of hives, sometimes with abdominal pain and diarrhoea, after cow’s milk exposure | 11mm | No | IgE CMA |
| 15 | Never exposed to cow’s milk. Avoided based on family history of cow’s milk allergy and personal history of other IgE mediated food allergies | 11mm | No | IgE CMA |
| 16 | Repeated episodes of hives after exposure to cow’s milk | 17mm | No | IgE CMA |
| 17 | Episode of pruritus after skin contact with cow’s milk, but never directly consumed due to other food allergies, eczema and positive allergy tests | 13mm at age 1 year | No | IgE CMA |
| 18 | Repeated episodes of hives with exposure to cow’s milk | 8mm | No | IgE CMA |
| 19 | Repeated episodes of rash within 30 minutes of exposure to cow’s milk | 6mm | Tolerating baked milk | IgE CMA |

# Table S4. Performance of screening questions in relation to confirmed primary care medical record of cow’s milk hypersensitivity

| BEEP Trial Screening Questions |  | Primary care record of cow’s milk hypersensitivity | Primary care record of cow’s milk hypersensitivity at <12 months | Low-allergy formula prescription recorded | Low-allergy formula prescription at <12 months |
| --- | --- | --- | --- | --- | --- |
| Answered ‘Yes’ to any of the 3 questions in records available for analysis (n=171) |  | 140/171 (82%) | 110/162 (68%) | 91/166 (55%) | 79/163 (48%) |
| Question 1: In the last year, has your baby had a reaction to any foods containing cow's milk protein? Asked at 12 months. | Yes (n=105)  No (n=34) | 91/105 (87%)  22/34 (65%) | 78/98 (80%)  9/34 (26%) | 61/102 (60%)  6/34 (18%) | 55/102 (54%)  5/34 (15%) |
| Question 2: Has your child ever had a reaction to foods containing cow’s milk? Asked at 24 months. | Yes (n=137)  No (n=27) | 116/137 (85%)  17/27 (63%) | 91/131 (69%)  13/24 (54%) | 78/134 (58%)  8/25 (32%) | 67/130 (52%)  7/26 (27%) |
| Question 3: In the last year, has your child been prescribed special low allergy formula milk (e.g. Nutramigen, Aptamil Pepti, Althera, Neocate)? Asked at 24 months. | Yes (n=54)  No (n=115) | 52/54 (96%)  86/115 (75%) | 46/52 (88%)  62/108 (57%) | 51/54 (94%)  40/110 (36%) | 44/50 (88%)  35/111 (32%) |

Comparison of parental responses to the 3 BEEP study screening questions with the information recorded in the analysed primary care records (n=171) related to primary care concern of reaction to milk and prescription of low-allergy formula (extensively hydrolysed, amino acid and soya). Denominators differ due to different numbers of participants with missing information for different variables.

# Table S5. Diagnostic process.

|  |  | **No confirmed CMA (n=124)** **N (%)** | | **Confirmed CMA (n=16)**  **N (%)** |
| --- | --- | --- | --- | --- |
| **Role of person who raised the initial concern of cow’s milk hypersensitivity** | General Practitioner  Parent†  Unclear  Secondary care specialist  Community HCP  Multiple | 39 (31.5%)  37 (29.8%)  14 (11.3%)  13 (10.5%)  11 (8.9%)  10 (8.1%) | 8 (50%)  4 (25%)  -  1 (6.3%)  1 (6.3%)  2 (12.5%) | |
| **Role of person who made the most definitive diagnosis of cow’s milk hypersensitivity** | Secondary care specialist  General Practitioner  Multiple  Dietician  Unclear  Parent  Community HCP | 44 (35.5%)  40 (32.3%)  17 (13.7%)  11 (8.9%)  9 (7.2%)  2 (1.6%)  1 (0.8%) | 7 (43.8%)  1 (6.3%)  8 (50%)  -  -  -  - | |
| **Classification of diagnostic label given** | Allergy  Non IgE-mediated  Intolerance  Unclear  IgE-mediated  Hypersensitivity | 54 (43.5%)  27 (21.8%)  26 (21%)  11 (8.9%)  3 (2.4%)  3 (2.4%) | 9 (56.3%)  -  -  -  7 (43.8%)  - | |
| **Nature of symptoms at initial concern of cow’s milk hypersensitivity‡** | Lower gastrointestinal  Upper gastrointestinal  Skin  Behavioural  Faltering Growth  Respiratory | 72 (58.1%)  62 (50%)  49 (39.5%)  29 (23.4%)  12 (9.7%)  8 (6.5%) | 7 (43.8%)  6 (37.5%)  15 (93.8%)  4 (25%)  1 (6.3%)  3 (18.8%) | |
| **Diagnostic assessments performed outside of the BEEP trial protocol** | None  Negative SPT/SIgE  Positive SPT/SIgE  Unknown  Supervised OFC | 89 (71.8%)  23 (18.5%)  6 (4.8%)  6 (4.8%)  0 | -  2 (12.5%)  8 (50%)  6 (37.5%)  0 | |

HCP, healthcare practitioner (usually health visitor); SPT, skin prick test.

†In just two primary care records (no confirmed CMA) a parent raised an initial concern of cow’s milk hypersensitivity but was advised by the GP that this diagnosis was unlikely. ‡Cutaneous symptoms alone were recorded for 17/124 (13.7%) no confirmed CMA and 6/16 (37.5%) confirmed CMA children, gastrointestinal symptoms alone 44/124 (35.5%) no confirmed CMA and 1/16 (6.3%) confirmed CMA, mixed symptoms in 61/124 (49.2%) no confirmed CMA and 9/16 (56.3%) confirmed CMA and unclear for 2 (1.6%) no confirmed CMA children. Mixed symptoms included symptoms from more than one system: gastrointestinal, cutaneous, faltering growth, respiratory and behavioural. Lower gastrointestinal symptoms included diarrhoea, constipation, mucus in stools, blood in stools, wind, and abdominal pain. Upper gastrointestinal symptoms included reflux and vomiting. Skin symptoms included rash, eczema, hives, urticaria, angioedema, and itch. Faltering growth included weight loss or drop in centiles. Respiratory symptoms included hoarseness, cough, floppy larynx, phlegm, breathing changes, wheeze, and choking. Behavioural symptoms included crying, colic, unsettled, food aversion, and sleep disturbance.

There was a statistically significant association between skin symptoms and CMA diagnosis (Chi-squared test, p<0.001, or p<0.006 adjusted for false discovery). No statistically significant difference was found between confirmed CMA and no confirmed CMA participants for lower gastrointestinal symptoms (p=0.246), upper gastrointestinal symptoms (p=0.288), behavioural (Fisher’s exact test, p=1.00), faltering growth (p=1.00) and respiratory symptoms (0.12).

# Table S6: Diet and Feeding

|  |  | **No confirmed CMA**  **n/N (%)** | **Confirmed CMA**  **n/N (%)** |
| --- | --- | --- | --- |
| **Feeding status at initial concern of cow’s milk hypersensitivity** | Breastfed, fully or partially  Not breastfed  Unclear | 53/124 (42.7%)  52/124 (41.9%)  19/124 (15.3%) | 12/16 (75%)  4/16 (25%)  - |
| **Feeding status at time of initial prescription of low-allergy formula** | Breastfed, fully or partially  Formula fed  Unclear | 38/81 (47%)  36/81 (44%)  7/81 (9%) | 7/10 (70%)  3/10 (30%)  - |
| **Maternal dietary restriction of dairy**† | Undertaken or advised  No record of relevant discussion  Unclear | 35/53 (66.0%)  16/53 (30.2%)  2/53 (3.8%) | 9/12 (75%)  3/12 (25%)  - |
| **Role of person who initiated or advised maternal dietary restriction** | Parent  Secondary Care Specialist  General Practitioner  Multiple  Community HCP  Unclear  Dietician | 18/35 (51.4%)  6/35 (17.1%)  4/35 (11.4%)  4/35 (11.4%)  2/35 (5.7%)  1/35 (2.9%)  - | 3/9 (33.3%)  1/9 (11.1%)  -  2/9 (22.2%)  -  -  3/9 (33.3%) |
| **Other dietary exclusion advised from maternal diet** | No record of soya exclusion  Soya exclusion  Unclear | 26/35 (74.3%)  7/35 (20.0%)  2/35 (5.7%) | 8/9 (88.9%)  -  1/9 (11.1%) |
| **Other dietary exclusion advised from child’s diet** | No record of soya exclusion  Soya exclusion  Unclear | 106/124 (85.5%)  9/124 (7.3%)  9/124 (7.3%) | 15/16 (93.8%)  1/16 (6.3%)  - |
| **Dietician review of child with possible milk reaction** | Yes  No  Unclear | 70/124 (56.5%)  44/124 (35.5%)  10/124 (8.1%) | 12/16 (75%)  4/16 (25%)  - |

†No record of advice against maternal dietary restriction. Denominators differ due to different numbers of participants with missing information for different variables.

Unclear refers to missing information. **No confirmed CMA BEEP cohort with a primary care concern of reaction to milk (n=124):** For role of person who initiated or advised maternal dietary restriction of dairy: Community healthcare practitioner (HCP) refers to Health Visitor (n=2); Multiple refers to GP and Health Visitor (n=1), GP and Paediatrician (n=1), Parent, Paediatrician and Paediatric Allergist (n=1), and Dietician, Paediatrician, and Paediatric Gastroenterologist (n=1); secondary care Specialist refers to Paediatrician (n=2), Paediatric Allergist (n=2), Dermatologist (n-1) and Paediatric Gastroenterologist (n=1). †One record of parental initiated maternal dietary restriction advised unnecessary by Paediatric Allergist. One record of initial GP advising maternal dietary restriction and another GP subsequently advising against maternal dietary restriction. **Confirmed CMA BEEP cohort (n=16):** For role of person who initiated or advised maternal dietary restriction of dairy: Multiple refers to parent and GP (n=1), and parent and paediatrician (n=1); secondary care Specialist refers to Paediatrician (n=1).

#

# Table S7: Low-allergy formula prescribed during the first year of life

|  |  | **No confirmed CMA**  **(n=81) N (%)** | **Confirmed CMA**  **(n=10) N (%)** |
| --- | --- | --- | --- |
| **Type of low-allergy formula first prescribed** | Extensively hydrolysed  Amino acid  Soya | 56/81 (69%)  17/81 (21%)  8/81 (10%) | 5/10 (50%)  4/10 (40%)  1/10 (10%) |
| **Role of person who initially advised, requested or prescribed low-allergy formula** | General Practitioner  Secondary care specialist  Dietician  Parent  Multiple  Unclear | 43/81 (53%)  21/81 (26%)  7/81 (9%)  4/81 (5%)  3/81 (4%)  3/81 (4%) | 4/10 (40%)  2/10 (30%)  2/10 (20%)  -  1/10 (10%)  - |
| **Indication for first low-allergy formula prescription** | Allergy  Intolerance  Non IgE-mediated  Other†  IgE-mediated  Unclear  Hypersensitivity | 47/81 (58%)  22/81 (27%)  4/81 (5%)  4/81 (5%)  2/81 (2%)  2/81 (2%)  - | 6/10 (60%)  1/10 (10%)  1/10 (10%)  -  2/10 (20%)  -  - |
| **Nature of symptoms at time of first low-allergy formula prescription‡** | Lower gastrointestinal  Upper gastrointestinal  Skin  Behavioural  Faltering Growth  Respiratory | 44/81 (54%)  37/81 (45%)  34/81 (42%)  25/81 (31%)  9/81 (11%)  1/81 (1%) | 4/10 (40%)  5/10 (50%)  9/10 (90%)  2/10 (20%)  1/10 (10%)  2/10 (20%) |
| **Second low-allergy formula prescribed** | No second formula  Amino Acid  Extensively hydrolysed  Soya | 47/81 (58%)  17/81 (21%)  14/81 (17%)  3/81 (4%) | 7/10 (70%)  1/10 (10%)  2/10 (20%)  - |
| **Third low-allergy formula prescribed** | No third formula  Amino acid  Extensively hydrolysed | 68/81 (84%)  9/81 (11%)  4/81 (5%) | 10/10 (100%)  -  - |
| **Other prescriptions used in participants prescribed low-allergy formula§** | Gaviscon® infant  Histamine 2 blocker  Proton Pump Inhibitor  Lactose free formula  Thickening agent  Colic medication | 35/81 (43%)  24/81 (30%)  12/81 (15%)  10/81 (12%)  7/81 (9%)  6/81 (7%) | 3/10 (30%)  1/10 (10%)  1/10 (10%)  1/10 (10%)  1/10 (10%)  - |

†Other refers to gastroesophageal disease (n=1), parent request (n=2), and primary intestinal lymphangiectasia (n=1).

‡Cutaneous symptoms only occurred in 12/81 (15%) participants, gastrointestinal symptoms only occurred in 20/81 (25%) participants, and mixed symptoms occurred in 44/81 (54%) participants. No symptoms were recorded at time of first prescription for 5/81 (6%) participants.

§ Antireflux medication only was prescribed to 8/81 (10%) participants without confirmed CMA, 2/10 (20%) confirmed CMA, multiple agents were prescribed to 33/81 (41%) participants without confirmed CMA, no additional medicines were prescribed to 33/81 (41%) participants without confirmed CMA and 5/10 (50%) confirmed CMA.

Skin symptoms were significantly more common in participants with confirmed CMA at time of first low-allergy formula prescription, compared with participants without confirmed CMA (p=0.006, or p=0.036 after adjustment for false discovery). No association was seen for frequency of lower gastrointestinal symptoms (p=0.308), upper gastrointestinal symptoms (p=1.00), behavioural (p=0.495), faltering growth (p=1.00) and respiratory symptoms (0.061; Fisher’s exact test).

#

# Table S8: Volume and cost of low-allergy formula prescribed to BEEP trial participants

#

|  | **No confirmed CMA (n=195)** | | | **Confirmed CMA diagnosis (n=19)** | | |
| --- | --- | --- | --- | --- | --- | --- |
|  | Participants included in analysis, n | Total,  n | Median (Q1, Q3) | Participants included in analysis, n | Total,  n | Median (Q1, Q3) |
| Total duration of low-allergy formula use (months)† | 68 | 792 | 10 (1,16) | 8 | 96 | 9 (3, 22) |
| Total volume of low-allergy formula (litres) | 68 | 20053 | 272 (26, 448) | 8 | 1748 | 182 (28, 389) |
| Total volume of ehf, aaf and soya (litres) | ehf (n=53)    aaf (n=34)    soya (n=2) | 7982    11951    119 | 42 (12, 248)    334 (122, 470)    60 (7, -) | ehf (n=6)    aaf (n=4)    soya (n=0) | 1329    419    - | 182 (47, 418)    46 (10, 259)  - |
| Total cost of low-allergy formula (£ sterling) | 68 | 119934 | 1214 (104, 2649) | 8 | 8497 | 854 (164, 1908) |
| Total cost of ehf, aaf and soya prescribed (£ sterling) | ehf (n=53)    aaf (n=34)    soya (n=2) | 29790    89898    246 | 160 (42, 864)    2504 (907, 3792)    123 (14, -) | ehf (n=6)    aaf (n=4)    soya (n=0) | 4952    3545    - | 689 (175, 1565)    520 (75, 2064)  - |

Low-allergy formula includes soya, extensively hydrolysed formula (ehf) and amino acid formula (aaf). Q1, 25^th^ centile. Q3, 75^th^ centile. Data analysis includes all ehf and aaf suitable for <1 year old and aaf suitable for >1 year old (Neocate Advance, Neocate Active and Neocate Junior). Volume in litres was calculated using product recommended dilution (%weight/volume equated to grams per 100mls).

†For those participants with missing data (age at final prescription) and once-off prescriptions, duration (in days, divided by 30 to convert to months) of low-allergy formula use was estimated according to volume of low-allergy formula prescribed and expected daily infant formula consumption of 780ml for infants aged <180 days old, 600ml for >/=180 days old at time of formula prescription. (8)

A once-off prescription was recorded for 14 participants (12 no confirmed CMA, 2 confirmed CMA) and ≥1 repeat prescription for >1 month was recorded for 58 participants (52 no confirmed CMA, 6 confirmed CMA). No significant differences were seen between no confirmed CMA and confirmed CMA groups in total months low-allergy formula (p=0.97), total volume prescribed (p=0.66), ehf volume (p=0.15), aaf volume (p=0.08), total cost of prescribed low-allergy formula (p=0.57), cost of ehf (p=0.15) and cost of aaf (p=0.08).

#

# Table S9: Estimations for incidence of cow’s milk hypersensitivity and low-allergy formula prescription in BEEP study cohort

|  | **Total BEEP cohort**  **(n=1394)** | | **BEEP excluding participants with confirmed CMA**  **(n=1375)** | |
| --- | --- | --- | --- | --- |
|  | Documented proportion | Estimated  n | Documented proportion  n | Estimated  n |
| Parent report of milk reaction | 214/(1394-166)=0.17 | (0.17x166)=29+214=243 | 195/1209=0.16 | (0.16x166)=27+195=222 |
| Primary care record of milk hypersensitivity | 140/171=0.82 | (0.82x43)=35+140=175 | 124/155=0.8 | (0.8x40)=32+124=156 |
| Low-allergy formula prescription | 91/(171-5)=0.55 | (0.55x(43+5)) + (0.55x (0.17x166))=27+15+91=133 | 81/(155-5)=0.54 | (0.54x(40+5)) + (0.54x (0.16x166)) =24+14+81=119 |

BEEP participants: 19/1394 with confirmed CMA, 195/1394 with CMA overdiagnosis, 1014/1394 assumed not to have CMA as parents answered no to screening questions.

Screening Questions for milk allergy: 214/1394 (yes), 1014/1394 (no), 166/ 1394 (never answered the questions)

Primary care records: 171/214 analysed, 43/214 unavailable records

Analysed primary care records: 5/171 prescription records missing (all CMA overdiagnosis), 16/171 confirmed CMA, 155/171 CMA overdiagnosis.

Unavailable primary care records: 40/43 CMA overdiagnosis, 3/43 confirmed CMA.

#

# Table S10: Method for calculating estimated volume and cost of low-allergy formula per infant born

|  | Proportion of analysed cohort with a parent reported milk reaction | Proportion of analysed parent reported CMA with a primary care prescription of low-allergy formula | Number of parent-reported CMA with known formula prescription information | Number of parent-reported CMA with missing formula prescription information | Number of formula prescribed with missing volume information | Estimated number of participants without information on CMA or prescription or volume who had a low-allergy formula prescription |
| --- | --- | --- | --- | --- | --- | --- |
| Total BEEP cohort | 0.17 | 0.55 | 76 | 48 | 15 | 56.9† |
| BEEP cohort excluding confirmed CMA | 0.16 | 0.54 | 68 | 45 | 13 | 51.6‡ |

BEEP participants who did not answer the screening questions = 166/1394

Number of parent-reported CMA with missing primary care records = 43 (40 CMA overdiagnosis, 3 confirmed CMA)

Number of parent-reported CMA with missing formula prescription information = 48 (43 missing primary care records and 5 missing prescription records)

†(166 no response to screening questions x 0.17 x 0.55) + (48 x 0.55) +15 = 56.9

‡ (166 no response to screening questions x 0.16 x 0.54) + (45 x 0.54) + 13 = 51.64

Documented volume and cost refer to the total sum observed in the primary care records.

# Table S11: Documented and estimated volume and cost of low-allergy formula in BEEP study cohort

|  | Documented volume (litres) | Estimated volume of low-allergy formula in participants without information on CMA, prescription or volume† | Total estimated volume of low-allergy formula | Total low-allergy formula volume per BEEP participant | Documented cost (sterling) | Estimated cost of low-allergy formula in participants without information on CMA, prescription or volume† | Total estimated cost of low-allergy formula | Total low-allergy formula cost per BEEP participant |
| --- | --- | --- | --- | --- | --- | --- | --- | --- |
| *Total BEEP cohort* | | | | | | | | |
| Any low-allergy formula suitable < 1 year old | 21654 | 16290 | 37944 | 27.22 | 125874.00 | 94689.67 | 220563.67 | 158.22 |
| eHF | 9311 | 7005 | 16316 | 11.70 | 34742.38 | 26135.21 | 60877.59 | 43.67 |
| AAF | 12223 | 9195 | 21418 | 15.36 | 90885.90 | 68369.59 | 159255.49 | 114.24 |
| Soya | 119 | 90 | 209 | 0.15 | 245.74 | 185.86 | 431.60 | 0.31 |
| *BEEP cohort excluding confirmed CMA* | | | | | | | | |
| Any low-allergy formula suitable < 1 year old | 19942 | 15179 | 35121 | 25.54 | 118003.30 | 89817.95 | 207875.25 | 151.14 |
| eHF | 7982 | 6076 | 14058 | 10.22 | 29790.00 | 22674.60 | 52464.60 | 38.16 |
| AAF | 11840 | 9012 | 20852 | 15.17 | 87967.44 | 66956.24 | 154923.68 | 112.67 |
| Soya | 119 | 91 | 210 | 0.15 | 245.74 | 187.04 | 432.78 | 0.31 |

Excluding products designed for age > 1 year old

†Calculated by estimated number of participants without information on CMA, prescription or volume who had a low-allergy formula prescription from Table 8b above (56.9 for total BEEP cohort and 51.64 for BEEP cohort excluding confirmed CMA) and considering the number of participants who had a documented volume prescribed in the primary care records

#

# Table S12: Volume and cost of low-allergy formula per infant born

|  | **England 2015†** | | **BEEP Primary Care Practices 2014** | | **BEEP cohort** | | **BEEP excluding infants with confirmed CMA** | |
| --- | --- | --- | --- | --- | --- | --- | --- | --- |
|  | Volume  (‘000 litres) | Volume per birth (L) | Volume  (‘000 litres) | Volume per birth (L) | Documented volume per birth (L) | Estimated volume per birth (L) | Documented volume per birth (L) | Estimated volume per birth (L) |
| Any prescribed low-allergy formula suitable for <1 year old ‡ | 9,611 | 14.47 | 837 | 13.56 | 15.53 | 27.22 | 14.50 | 25.54 |
| Extensively hydrolysed formula | 5,863 | 8.82 | 487 | 7.88 | 6.68 | 11.70 | 5.81 | 10.22 |
| Amino acid formula | 3,234 | 4.87 | 297 | 4.81 | 8.77 | 15.36 | 8.61 | 15.17 |
| Soya formula | 513 | 0.77 | 53 | 0.86 | 0.09 | 0.15 | 0.09 | 0.15 |
|  | Cost (£ thousand) | Cost per birth (£) | Cost (£ thousand) | Cost per birth (£) | Documented cost per birth (£) | Estimated cost per birth (£) | Documented cost per birth (£) | Estimated cost per birth (£) |
| Any prescribed low-allergy formula suitable for <1 year old‡ | 51,424 | 77.40 | - | - | 90.30 | 158.22 | 85.82 | 151.14 |
| Extensively hydrolysed formula | 20,468 | 30.81 | - | - | 24.92 | 43.67 | 21.67 | 38.16 |
| Amino acid formula | 30,228 | 45.50 | - | - | 65.20 | 114.24 | 63.98 | 112.67 |
| Soya formula | 728 | 1.10 | - | - | 0.18 | 0.31 | 0.18 | 0.31 |

†Data for quantity (grams) and cost (UK pounds sterling) of low-allergy formula prescribed in England in 2015 was obtained from the National Health Service Prescription Cost Analysis reports; live birth rate for England for 2015 (664,399 births) was obtained from the UK Office of the National Statistics; volumes in litres were calculated using product recommended dilution. (9) ‡Products excluded from analysis include Neocate Advance, Neocate Active and Neocate Junior. One participant from the no confirmed CMA cohort who had quantity of formula recorded in the notes was prescribed aaf over the age of 12 months, but all products included in both the 2015 national analysis and the BEEP study analysis are products designed for use in infants age <12 months Products marketed for children over age 12 months were prescribed to five BEEP participants with CMA overdiagnosis (Neocate Advance n=2, Neocate Junior n=2, Neocate Active n=1) and one BEEP participant with confirmed CMA (Neocate Active) over 12 months old. Estimations assume the same proportion of parent-reported milk reaction, primary care record of cow’s milk hypersensitivity or primary care record of low-allergy formula prescription in the participants with unavailable records or missing information.

# Table S13: Prescription of extensively hydrolysed formula and amino acid formula in BEEP cohort

|  | Participants included in analysis | Median (Q1, Q3) |
| --- | --- | --- |
| Age at first prescription (Days) | ehf (n=64)    aaf (n=39)    aaf suitable >1year (n=6) | 141 (70, 236)    115 (60, 155)    404 (368, 510) |
| Age at final prescription (Days) | ehf (n=56)    aaf (n=37)    aaf suitable >1year (n=6) | 288 (106, 573)    390 (279, 539)    455 (368, 543) |
| Duration of low-allergy formula (Days)† | ehf (n=59)    aaf (n=37)  aaf suitable >1year (n=6) | 64 (15, 373)    275 (115, 437)  32 (14, 61) |
| Duration of low-allergy formula prescribed excluding once-off prescriptions (Days) | ehf (n=36)    aaf (n=29)    aaf suitable >1year (n=2) | 308 (94, 486)    335 (272, 483)    73 (42, -) |
| Volume / Day (Litres)‡ | ehf (n=36)    aaf (n=29)    aaf suitable >1year (n=2) | 0.61 (0.31, 1.04)    1.01 (0.77, 1.20)    0.37 (0.09, -) |

ehf, extensively hydrolysed formula; aaf, amino acid formula (aaf). Q1, 25^th^ centile. Q3, 75^th^ centile. †For those participants with once-off prescriptions and with missing data for age at final prescription, duration (days) of low-allergy formula use was estimated according to volume of low-allergy formula prescribed and expected daily infant formula consumption of 780ml for infants aged <180 days old, 600ml for >/=180 days old and 400ml for infants aged =/> 365 days old at time of prescription of formula. (8,10) ‡Volume / Day refers to volume prescribed from initial prescription to final prescription excluding once-off prescriptions and excluding the volume prescribed at the final prescription.

#

# Table S14: Repeat prescriptions of extensively hydrolysed formula and amino acid formula in BEEP cohort categorising participants according to the main formula prescribed

|  | Participants included in analysis  (mainly ehf, n=21; mainly aaf, n=27) | Median (Q1, Q3) |
| --- | --- | --- |
| Age at first prescription (Days) | ehf    aaf | 168 (86, 227)    84 (56, 119) |
| Age at final prescription (Days) | ehf    aaf | 568 (403, 879)    437 (345, 579) |
| Duration of low-allergy formula prescribed excluding once-off prescriptions and breastfeeding participants using top-up formula (Days) | ehf    aaf | 429 (308, 684)    358 (272, 496) |
| Volume / Day excluding once-off prescriptions and breastfeeding participants using top-up formula (Litres) | ehf    aaf | 0.64 (0.42, 0.89)    1.01 (0.86, 1.20) |
| Exclusively formula fed from birth | ehf 6/25 (24%)  aaf 6/29 (20.7%) |  |

ehf, extensively hydrolysed formula; aaf, amino acid formula (aaf). Q1, 25^th^ centile. Q3, 75^th^ centile. Participants were categorised into mainly ehf (n=25) or aaf (n=29) determined by longest duration of use of each formula. Once-off prescriptions, volume of final prescription and low volumes used for top-up formula with breastfeeding (mainly ehf, n=4; mainly aaf, n=2) were excluded from analysis. This ensured that only participants receiving repeat prescriptions of known volume and for known duration were analysed. A Mann-Whitney U test (n=48) was undertaken to determine differences between BEEP participants prescribed mainly extensively hydrolysed and mainly amino acid formula suitable for <1 year old. A statistically significant difference was found between participants prescribed mainly ehf or mainly aaf for age at first prescription (p=0.004, U=423) and volume consumed per day (p=0.001, U=126). No statistically significant difference was found for age at final prescription (p=0.07, U= 371) or duration of use (p=0.143, U=354).

# Table S15: Participant characteristics and parent-reported cow’s milk reaction

|  | **Unadjusted** | | **Adjusted** (n=777)† | | **Adjusted** (n=777)‡ | |
| --- | --- | --- | --- | --- | --- | --- |
|  | **OR**  **(95% CI)** | **P-value** | **OR**  **(95% CI)** | **P-value** | **OR**  **(95% CI)** | **P-value** |
| **Maternal Age** (n=1209) | 0.99  (0.96-1.02) | 0.35 | 0.99  (0.95-1.03) | 0.67 | - | - |
| **White maternal ethnicity** (white, n=1037; non-white, n=172) | 1.04  (0.67-1.62) | 0.87 | 0.85  (0.45-1.63) | 0.64 | - | - |
| **Antibiotics used in pregnancy**  (yes, n=345; no, n=854) | 1.58  (1.15-2.19) | 0.005 | 1.70  (1.12-2.59) | 0.01 | 1.79  (1.19-2.70) | 0.006 |
| **More than one first degree relative with atopic disease**  (two or more, n=767; one, n=442) | 1.04  (0.75-1.42) | 0.83 | 0.92  (0.59-1.43) | 0.71 | - | - |
| **No other children in household**  (only child, n=506; other children, n=703) | 0.96  (0.70-1.31) | 0.80 | 0.91  (0.59-1.40) | 0.66 | - | - |
| **Maternal anxiety/depression on EQ-5D (at baseline)**  (any anxiety, n=188; no anxiety, n=709) | 0.89  (0.57-1.39) | 0.59 | 0.70  (0.41-1.20) | 0.19 | - | - |
| **Maternal EQ-5D health state at baseline** (n=891) | 0.99  (0.98-1.00) | 0.08 | 0.99  (0.97-1.00) | 0.11 | - | - |
| **Exclusive formula feeding from birth to 6 months old**  (formula only, n=157; all other feeding, n=837) | 1.53  (1.00-2.35) | 0.05 | 1.50  (0.89-2.52) | 0.13 | - | - |
| **Family decile of English Index of Multiple Deprivation 2015**  (n=1185) | 0.97  (0.92-1.03) | 0.34 | 1.00  (0.93-1.08) | 0.97 | - | - |

Logistic regression comparing participants with a positive response to one of the 3 screening questions excluding the 19 confirmed milk allergic participants (n=195) with the remaining BEEP study cohort who answered the screening questions (n=1014). BEEP participants who did not answer the screening questions were excluded from analysis (n=166). Odds ratio (OR) >1 shows a positive association between each variable and parental reported reaction to milk or low-allergy formula prescription (outcome). Confidence intervals are 95% and p-value <0.05 indicates statistical significance. Unadjusted values indicate an association alone and adjusted values (n=777) consider the association of all variables together on the outcome. †Step 1 of backward stepwise regression including all co-variates ‡Final step of backward stepwise regression

**Table S16: Participant characteristics and primary care record of cow’s milk hypersensitivity**

|  | **Unadjusted** | | **Adjusted** (n=758)† | | **Adjusted** (n=758)‡ | |
| --- | --- | --- | --- | --- | --- | --- |
|  | **OR**  **(95% CI)** | **P-value** | **OR**  **(95% CI)** | **P-value** | **OR**  **(95% CI)** | **P-value** |
| **Maternal Age** (n=1169) | 0.99  (0.95-1.02) | 0.45 | 0.99  (0.94-1.04) | 0.68 | - | - |
| **White maternal ethnicity** (white, n=1002; non-white, n=167) | 0.98  (0.58-1.67) | 0.94 | 0.61  (0.30-1.24) | 0.17 | - | - |
| **Antibiotics used in pregnancy**  (yes, n=331; no, n=828) | 1.91  (1.30-2.80) | <0.001 | 2.00  (1.22-3.28) | 0.006 | 2.11  (1.30-3.42) | 0.003 |
| **More than one first degree relative with atopic disease**  (two or more, n=742; one, n=427) | 1.14  (0.77-1.69) | 0.52 | 0.89  (0.53-1.51) | 0.67 | - | - |
| **No other children in household**  (only child, n=486; other children, n=683) | 0.84  (0.57-1.24) | 0.38 | 0.83  (0.49-1.40) | 0.48 | - | - |
| **Maternal anxiety/depression on EQ-5D (at baseline)**  (any anxiety, n=184; no anxiety, n=686) | 0.98  (0.58-1.65) | 0.94 | 0.81  (0.43-1.52) | 0.52 | - | - |
| **Maternal EQ-5D health state at baseline** (n=863) | 0.99  (0.97-1.01) | 0.17 | 0.98  (0.97-1.00) | 0.09 | - | - |
| **Exclusive formula feeding from birth to 6 months old**  (formula only, n=151; all other feeding, n=814) | 1.78  (1.09-2.93) | 0.02 | 1.79  (0.98-3.25) | 0.06 | 1.72  (0.96-3.09) | 0.07 |
| **Family decile of English Index of Multiple Deprivation 2015** (n=1146) | 0.97  (0.90-1.04) | 0.35 | 1.03  (0.94-1.13) | 0.56 | - | - |

Logistic regression comparing potential risk factors associated with a positive mention of reaction to milk in the primary care records (n=124) excluding the 19 confirmed milk allergic participants. Participants who did not answer yes to any of the screening questions were assumed not to have a reaction to milk (n=1045). Participants who did not answer any of the screening questions (n=166) and the primary care records that were not received (n=40) were excluded from analysis. Odds ratio (OR) >1 shows a positive association between each variable and a reaction to milk in the records (outcome). Confidence intervals are 95% and p-value <0.05 indicates statistical significance. Unadjusted values indicate an association alone and adjusted values (n=758) consider the association of all variables together on the outcome. †Step 1 of backward stepwise regression including all co-variates ‡Final step of backward stepwise regression

**Table S17: Participant characteristics and primary care record of low-allergy formula prescription**

|  | **Unadjusted** | | **Adjusted** (n=757)† | | **Adjusted** (n=757)‡ | |
| --- | --- | --- | --- | --- | --- | --- |
|  | **OR**  **(95% CI)** | **P-value** | **OR**  **(95% CI)** | **P-value** | **OR**  **(95% CI)** |  |
| **Maternal Age** (n=1164) | 0.97  (0.92-1.01) | 0.10 | 0.96  (0.91-1.02) | 0.24 | - | - |
| **White maternal ethnicity** (white, n=999; non-white, n=165) | 1.19  (0.60-2.35) | 0.63 | 0.78  (0.31-1.97) | 0.60 | - | - |
| **Antibiotics used in pregnancy**  (yes, n=330; no, n=824) | 2.05  (1.30-3.26) | 0.002 | 2.25  (1.25-4.05) | 0.007 | 2.36  (1.33-4.18) | 0.003 |
| **More than one first degree relative with atopic disease**  (two or more, n=738; one, n=426) | 1.24  (0.77-2.01) | 0.38 | 1.11  (0.59-2.09) | 0.76 | - | - |
| **No other children in household**  (only child, n=486; other children, n=678) | 0.96  (0.60-1.51) | 0.85 | 1.10  (0.59-2.04) | 0.77 | - | - |
| **Maternal anxiety/depression on EQ-5D (at baseline)**  (any anxiety, n=184; no anxiety, n=684) | 1.00  (0.54-1.85) | 1.00 | 0.87  (0.41-1.84) | 0.72 | - | - |
| **Maternal EQ-5D health state at baseline** (n=861) | 0.99  (0.97-1.01) | 0.46 | 0.99  (0.97-1.01) | 0.46 | - | - |
| **Exclusive formula feeding from birth to 6 months old**  (formula only, n=150; all other feeding, n=811) | 2.32  (1.31-4.13) | 0.004 | 2.51  (1.30-4.86) | 0.006 | 2.50  (1.31-4.75) | 0.005 |
| **Family decile of English Index of Multiple Deprivation 2015** (n=1142) | 0.97  (0.89-1.05) | 0.39 | 1.02  (0.91-1.15) | 0.68 | - | - |

Logistic regression comparing potential risk factors associated with a prescription of low-allergy formula in the primary care records (n=77) excluding the 19 confirmed milk allergic participants. Participants who did not answer yes to any of the screening questions were assumed not to have received a prescription (n=1086). Participants who did not answer any of the screening questions (n=166), the primary care records that were not received (n=40), and the participants with a positive mention of a reaction to milk whose prescription records were missing (n=5) were excluded from analysis. Odds ratio (OR) >1 shows a positive association between each variable and a low-allergy formula prescription (outcome). Confidence intervals are 95% and p-value <0.05 indicates statistical significance. Unadjusted values indicate an association alone and adjusted values (n=757) consider the association of all variables together on the outcome. †Step 1 of backward stepwise regression including all co-variates ‡Final step of backward stepwise regression.

# Table S18: Participant characteristics and primary care record of a low-allergy formula prescription in participants with a primary care record of cow’s milk hypersensitivity

|  | **Unadjusted** | | **Adjusted** (n=72)† | | **Adjusted** (n=72)‡ | |
| --- | --- | --- | --- | --- | --- | --- |
|  | **OR**  **(95% CI)** | **P-value** | **OR**  **(95% CI)** | **P-value** | **OR**  **(95% CI)** | **P-value** |
| **Maternal Age** (n=119) | 0.96  (0.89-1.03) | 0.24 | 0.84  (0.71-0.99) | 0.03 | 0.85  (0.74-0.97) | 0.02 |
| **White maternal ethnicity** (white, n=103; non-white, n=16) | 1.27  (0.42-3.80) | 0.67 | 1.01  (0.16-6.44) | 0.99 | - | - |
| **Antibiotics used in pregnancy (**yes, n=50; no, n=68) | 1.09  (0.50-2.37) | 0.84 | 0.87  (0.19-4.01) | 0.86 | - | - |
| **No other children in household** (only child, n=47; other children, n=72) | 1.07  (0.49-2.34) | 0.87 | 0.94  (0.20-4.51) | 0.94 | - | - |
| **Maternal anxiety/depression on EQ-5D (at baseline)** (any anxiety, n=20; no anxiety, n=74) | 1.05  (0.36-3.09) | 0.93 | 3.96  (0.42-37.47) | 0.23 | - | - |
| **Maternal EQ-5D health state (at baseline)** (n=94) | 1.01  (0.98-1.05) | 0.52 | 1.01  (0.96-1.06) | 0.76 | - | - |
| **Family decile of English Index of Multiple Deprivation 2015** (n=115) | 0.97  (0.84-1.12) | 0.66 | 0.86  (0.65-1.14) | 0.29 | - | - |
| **Age of child when symptoms were first considered related to milk (weeks)** (n=113) | 0.96  (0.94-0.98) | <0.001 | 0.97  (0.95-1.00) | 0.04 | 0.97  (0.94-1.00) | 0.02 |
| **Formula fed only at first mention of milk related symptoms**  (formula only, n= 49; all other feeding, n=55) | 0.77  (0.32-1.86) | 0.57 | 1.30  (0.31-5.38) | 0.72 | - | - |
| **Parent only raised the initial concern about reaction to milk** (parent only, n=36; healthcare practitioner with/without parent, n=72) | 0.62  (0.27-1.41) | 0.25 | 1.32  (0.30-5.81) | 0.72 | - | - |

Multivariate logistic regression comparing potential risk factors associated with a prescription of low-allergy formula within the cohort who have a positive mention of a reaction to milk in the primary care records, excluding the 19 confirmed milk allergic participants (n=124). Prescription records were missing for 5 participants with a reported reaction to milk in the primary care records. Odds ratio (OR) >1 shows a positive association between each variable and a low-allergy formula prescription (outcome). Confidence intervals are 95% and p-value <0.05 indicates statistical significance. Unadjusted values indicate an association alone and adjusted values (n=72) consider the association of all variables together on the outcome. †Step 1 of backward stepwise regression including all co-variates ‡Final step of backward stepwise regression

#

# Table S19. Missingness of participant-level predictors and CMA outcome variables in the BEEP trial dataset

| **Predictor and outcome variables** | **Missing data** |
| --- | --- |
| EQ5D health state at baseline (answered antenatally or shortly after birth) | 445/1394 (31.9%) |
| EQ5D anxiety at baseline (answered antenatally or shortly after birth) | 439/1394 (31.5%) |
| Infant feeding status from birth to 6 months | 366/1394 (26.3%) |
| Decile of English Index of Multiple Deprivation 2015 | 26/1394 (1.9%) |
| Maternal use of antibiotics in pregnancy | 12/1394 (0.9%) |
| Parent reported reaction to milk | 166/1394 (11.9%) |
| Primary care record of milk reaction | 209/1394 (15%) |
| Primary care record of low-allergy formula prescription | 214/1394 (15.4%) |

# Table S20. Characteristics of BEEP study participants without confirmed CMA, comparing complete versus missing data for predictor variables with high levels of missingness.

|  |  | **EQ-5D Any maternal anxiety at baseline** | | **EQ-5D maternal Health state at baseline** | | **Exclusive formula feeding from birth-6months** | |
| --- | --- | --- | --- | --- | --- | --- | --- |
|  |  | **Complete**  **(n=897)** | **Missing**  **(n=312)** | **Complete**  **(n=891)** | **Missing**  **(n=318)** | **Complete**  **(n=994)** | **Missing**  **(n=215)** |
| **Maternal Age**  (n=1209) | Mean  Std Dev  Median  (Q1, Q3) | 32.37  4.832  33  (29, 36) | 30.91  5.469  32  (27, 35) | 32.34  4.826  32  (29, 36) | 31.03  5.499  32  (27, 35) | 32.27  4.79  32.50  (29, 36) | 30.70  5.919  31  (26, 35) |
| **White maternal ethnicity**  (n=1209) | White  Non-White | 804 (89.6%)  93 (10.4%) | 233 (74.7%)  79 (25.3%) | 799 (89.7%)  92 (10.3%) | 238 (74.8%)  80 (25.2%) | 876 (88.1%)  118 (11.9%) | 161 (74.9%)  54 (25.1%) |
| **Antibiotics used in pregnancy**  (n=1199, missing =10) | Yes  No  Unknown | 254 (28.3%)  636 (70.9%)  7 (0.8%) | 91 (29.2%)  218 (69.9%)  3 (1%) | 251 (28.2%)  633 (71%)  7 (0.8%) | 94 (29.6%)  221 (69.5%)  3 (0.9%) | 273 (27.5%)  711 (71.5%)  10 (1%) | 72 (33.5%)  143 (66.5%) |
| **More than one first degree relative with atopic disease**  (n=1209) | Two or more  One | 554 (61.8%)  343 (38.2%) | 213 (68.3%)  99 (31.7%) | 551 (61.8%)  340 (38.2%) | 216 (67.9%)  102 (32.1%) | 623 (62.7%)  371 (37.3%) | 144 (67%)  71 (33%) |
| **No other children in household**  (n=1209**)** | Other children  Only Child | 507 (56.5%)  390 (43.5%) | 196 (62.8%)  116 (37.2%) | 503 (56.5%)  388 (43.5%) | 200 (62.9%)  118 (37.1%) | 550 (55.3%)  444 (44.7%) | 153 (71.2%)  62 (28.8%) |
| **Maternal anxiety/depression on EQ-5D (at baseline)**  (n=897, missing=312) | No anxiety  Any anxiety  Unknown | 709 (79%)  188 (21%)  - | -  -  312 (100%) | 702 (78.8%)  187 (21%)  2 (0.2%) | 7 (2.2%)  1 (0.3%)  310 (97.5%) | 164 (16.5%)  643 (64.7%)  187 (18.8%) | 66 (30.7%)  24 (11.2%)  125 (58.1%) |
| **Maternal EQ-5D health state at baseline**  (n=891, missing=318) | Mean  Std Dev  Median  (Q1, Q3) | 79.85  12.87  80  (70, 90) | 60  28.284  60  (40, -) | 79.81  12.94  80  (70, 90) | -  -  -  - | 79.92  12.851  80  (70, 90) | 78.79  13.74  80  (70, 90) |
| **Exclusive formula feeding from birth to 6 months old**  (n=994, missing =215) | Yes  No  Unknown | 118 (13.2%)  689 (76.8%)  90 (10%) | 39 (12.5%)  148 (47.4%)  125 (40.1%) | 117 (13.1%)  684 (76.8%)  90 (10.1%) | 40 (12.6%)  153 (48.1%)  125 (39.3%) | 157 (15.8%)  837 (84.2%)  - | -  -  215 (100%) |
| **Family decile of English Index of Multiple Deprivation 2015**  (n=1185, missing=24) | Mean  Std Dev  Median  (Q1, Q3) | 6.33  2.70  6  (4, 9) | 5.17  2.883  5  (3, 8) | 6.33  2.698  6  (4, 9) | 5.19  2.895  5  (3, 8) | 6.28  2.717  6  (4, 9) | 4.89  2.868  5  (2, 7) |
| **Parent-reported CMA (Positive response to any of screening questions)** (n=1209) | Yes  No | 145 (16.2%)  752 (83.8%) | 50 (16%)  262 (84%) | 146 (16.4%)  745 (83.6%) | 49 (15.4%)  269 (84.6%) | 157 (15.8%)  837 (84.2%) | 38 (17.7%)  177 (82.3%) |
| **Primary care record of reaction to milk** (yes=124, no=31, records not analysed =1054) | Yes  No  No primary care records available  No parental reported milk reaction | 96 (10.7%)  22 (2.5%)  27 (3%)  752 (84%) | 28 (9%)  9 (2.9%)  13 (4%)  262 (84%) | 96 (10.8%)  22 (2.5%)  28 (3%)  745 (84%) | 28 (8.8%)  9 (2.8%)  12 (4%)  269 (85%) | 102 (10.3%)  26 (2.6%)  29 (3%)  837 (84%) | 22 (10.2%)  5 (2.3%)  11 (5%)  177 (82%) |
| **Primary care record of low-allergy formula prescription (**yes=81, no=69, unknown as records not analysed=1054 | Yes  No  No primary care records available  No parental reported milk reaction | 66 (7.4%)  50 (5.6%)  29 (3%)  752 (84%) | 15 (4.8%)  19 (6.1%)  16 (5%)  262/312 (84%) | 66 (7.4%)  50 (5.6%)  30 (3%)  745 (84%) | 15 (4.7%)  19 (6%)  15 (5%)  269 (85%) | 63 (6.35)  61 (6.1%)  33 (3%)  837 (84%) | 18 (8.4%)  8 (3.7%)  12 (6%)  177 (82%) |

EQ-5D, standardised measure of health related quality of life. Questionnaire was completed during the BEEP trial when the mother was enrolled

Participants included in analysis = 1209/1394. BEEP participants who did not answer the screening questions were excluded from analysis (n=166) and the 19 confirmed milk allergic participants. Low-allergy formula prescription includes extensively hydrolysed, amino acid and soya formula.

# Table S21. Characteristics of BEEP participants with confirmed CMA, comparing complete versus missing data for predictor variables with high levels of missingness.

|  |  | **EQD5 Any anxiety0** | | **EDQ5 Health0** | | **Formula fed only at first mention milk related symptoms** | | **Parent only raised the initial concern about reaction to milk** | |
| --- | --- | --- | --- | --- | --- | --- | --- | --- | --- |
|  |  | **Complete**  **(n=94)** | **Missing**  **(n=25)** | **Complete**  **(n=94)** | **Missing**  **(n=25)** | **Complete**  **(n=104)** | **Missing**  **(n=15)** | **Complete (n=108)** | **Missing (n=11)** |
| Maternal Age  (n=119) | Mean  Std Dev  Median  (Q1, Q3) | 32.05  5.387  33  (29, 36) | 29.48  4.762  31  (27.50, 33) | 32.05  5.387  33  (29, 36) | 29.48  4.762  31  (27.50, 33) | 31.53  5.386  32  (29, 35) | 31.40  5.248  33  (30, 35) | 31.68  5.099  32  (29, 35) | 29.91  7.489  30  (24, 36) |
| White maternal ethnicity  (n=119) | White  Non-White | 81 (86.2%)  13 (13.8%) | 22 (88%)  3 (12%) | 81 (86.2%)  13 (13.8%) | 22 (88%)  3 (12%) | 91 (87.5%)  13 (12.5%) | 12 (80%)  3 (20%) | 95 (88%)  13 (12%) | 8 (72.7%)  3 (27.3%) |
| Antibiotics used in pregnancy  (n=118, missing =1) | Yes  No  Unknown | 38 (40.4%)  55 (58.5%)  1 (1.1%) | 12 (48%)  13 (52%)  0 | 38 (40.4%)  55 (58.5%)  1 (1.1%) | 12 (48%)  13 (52%)  0 | 45 (43.3%)  58 (55.8%)  1 (1%) | 5 (33.3%)  10 (66.7%)  0 | 46 (42.6%)  61 (56.5%)  1 (0.9%) | 4 (36.4%)  7 (63.6%)  0 |
| No other children in household  (n=119) | Other children  Only Child | 56 (59.6%)  38 (40.4%) | 16 (64%)  9 (36%) | 56 (59.6%)  38 (40.4%) | 16 (64%)  9 (36%) | 62 (59.6%)  42 (59.6%) | 10 (66.7%)  5 (33.3%) | 65 (60.2%)  43 (39.8%) | 7 (63.6%)  4 (36.4%) |
| Maternal anxiety/depression on EQ-5D (at baseline)  (n=94, missing=25) | No anxiety  Any anxiety  Unknown | 74 (78.7%)  20 (21.3%)  - | -  -  25 (100%) | 74 (78.7%)  20 (21.3%)  0 | -  -  25 (100%) | 65 (62.5%)  16 (15.4%)  23 (22.1%) | 9 (60%)  4 (26.7%)  2 (13.3%) | 67 (62%)  19 (17.6%)  22 (20.4%) | 7 (63.6%)  1 (9.1%)  3 (27.3%) |
| Maternal EQ-5D health state at baseline (n=94, missing=25) | Mean  Std Dev  Median  (Q1, Q3) | 78.31  12.44  80  (70, 90) | -  -  -  - | 78.31  12.44  80  (70, 90) | -  -  -  - | 78.41  12.75  80  (70, 90) | 77.69  10.727  80  (67.50, 85) | 78.33  12.58  80  (70, 90) | 78.13  11.63  30  (70, 91.25) |
| Family decile of English Index of Multiple Deprivation 2015  (n=115, missing=4) | Mean  Std Dev  Median  (Q1, Q3) | 6.13  5.387  33  (29, 36) | 4.76  2.773  4  (2.50, 7) | 6.13  2.687  6  (4, 9) | 4.76  2.773  4  (2.50, 7) | 5.86  2.72  5  (4, 9) | 5.64  3.079  6  (2.75, 8.25) | 5.98  2.75  6  (4, 9) | 4.3  2.452  3  (2.75, 5.75) |
| Age of child when symptoms were first considered related to milk (weeks) (n=113, missing=6) | Mean  Std Dev  Median  (Q1, Q3) | 217.93  196.75  169  (76, 300) | 196.22  211.64  142  (54, 256) | 217.93  196.75  169  (76, 300) | 196.22  211.64  142  (54, 256) | 178.56  159.98  137  (57, 231) | 537.64  239.63  462  (311, 712) | 216.30  203.23  168  (62, 300) | 163.83  95.56  169.50  (60, 244.50) |
| Formula fed only at first mention of milk related symptoms  (n=104, missing=15) | Yes  No  Unknown | 34 (36.2%)  47 (50%)  13 (13.8%) | 15 (60%)  8 (32%)  2 (8%) | 34 (36.2%)  47 (50%)  13 (13.8%) | 15 (60%)  8 (32%)  2 (8%) | 55 (52.9%)  49 (47.1%)  - | -  -  15 (100%) | 46 (42.6%)  52 (48.1%)  10 (9.3%) | 3 (37.2%)  3 (27.3%)  5 (45.5%) |
| Parent only raised the initial concern about reaction to milk (n=108, missing n=11) | Yes  No  Unknown | 28 (29.8%)  58 (61.7%)  8 (8.5%) | 8 (32%)  14 (56%)  3 (12%) | 28 (29.8%)  58 (61.7%)  8 (8.5%) | 8 (32%)  14 (56%)  3 (12%) | 30 (28.8%)  68 (65.4%)  6 (5.8%) | 6 (40%)  4 (26.7%)  5 (33.3%) | 36 (33.3%)  72 (66.7%)  - | -  -  11 (100%) |
| Primary care record of reaction to milk (n=119) | Yes  No  Unknown | 94 (100%)  0  0 | 25 (100%)  0  0 | 94 (100%)  0  0 | 25 (100%)  0  0 | 104 (100%)  0  0 | 15 (100%)  0  0 | 108 (100%)  0  0 | 11 (100%)  0  0 |
| Primary care record of low-allergy formula prescription (n=119) | Yes  No  Unknown | 65 (69.1%)  29 (30.9%)  0 | 15 (60%)  10 (40%)  0 | 65 (69.1%)  29 (30.9%)  0 | 15 (60%)  10 (40%)  0 | 77 (74%)  27 (26%)  0 | 3 (20%)  12 (80%)  0 | 71 (65.7%)  37 (34.3%)  0 | 9 (81.8%)  2 (18.2%)  0 |

Participants included in analysis = 119. BEEP participants with confirmed milk allergy (n=19) and participants with a milk reaction in primary care records but missing prescription records (n=5) were excluded. Low-allergy formula prescription includes extensively hydrolysed, amino acid and soya formula.

# Table S22: Participant characteristics and parent-reported cow’s milk reaction (excluding EQ5D)

|  | **Unadjusted** | | **Adjusted** (n=963)† | | **Adjusted** (n=963)‡ | |
| --- | --- | --- | --- | --- | --- | --- |
|  | **OR**  **(95% CI)** | **P-value** | **OR**  **(95% CI)** | **P-value** | **OR**  **(95% CI)** | **P-value** |
| **Maternal Age** (n=1209) | 0.99  (0.96-1.02) | 0.35 | 0.99  (0.96-1.04) | 0.96 | - | - |
| **White maternal ethnicity** (white, n=1037; non-white, n=172) | 1.04  (0.67-1.62) | 0.87 | 1.06  (0.60-1.85) | 0.85 | - | - |
| **Antibiotics used in pregnancy**  (yes, n=345; no, n=854) | 1.58  (1.15-2.19) | 0.005 | 1.72  (1.19-2.48) | 0.004 | 1.72  (1.19-2.48) | 0.004 |
| **More than one first degree relative with atopic disease**  (two or more, n=767; one, n=442) | 1.04  (0.75-1.42) | 0.83 | 0.97  (0.66-1.42) | 0.86 | - | - |
| **No other children in household**  (only child, n=506; other children, n=703) | 0.96  (0.70-1.31) | 0.80 | 0.97  (0.66-1.42) | 0.87 | - | - |
| **Exclusive formula feeding from birth to 6 months old**  (formula only, n=157; all other feeding, n=837) | 1.53  (1.00-2.35) | 0.05 | 1.49  (0.96-2.32) | 0.08 | 1.51  (0.97-2.34) | 0.07 |
| **Family decile of English Index of Multiple Deprivation 2015**  (n=1185) | 0.97  (0.92-1.03) | 0.34 | 0.99  (0.93-1.06) | 0.75 | - | - |

Logistic regression comparing participants with a positive response to one of the 3 screening questions excluding the 19 confirmed milk allergic participants (n=195) with the remaining BEEP study cohort who answered the screening questions (n=1014). BEEP participants who did not answer the screening questions were excluded from analysis (n=166). Odds ratio (OR) >1 shows a positive association between each variable and parental reported reaction to milk or low-allergy formula prescription (outcome). Confidence intervals are 95% and p-value <0.05 indicates statistical significance. Unadjusted values indicate an association alone and adjusted values (n=963) consider the association of all variables together on the outcome. †Step 1 of backward stepwise regression including all co-variates ‡Final step of backward stepwise regression

# Table S23. Participant characteristics and primary care record of cow’s milk hypersensitivity (excluding EQ5D)

|  | **Unadjusted** | | **Adjusted** (n=935)† | | **Adjusted** (n=935)‡ | |
| --- | --- | --- | --- | --- | --- | --- |
|  | **OR**  **(95% CI)** | **P-value** | **OR**  **(95% CI)** | **P-value** | **OR**  **(95% CI)** | **P-value** |
| **Maternal Age** (n=1169) | 0.99  (0.95-1.02) | 0.45 | 0.99  (0.95-1.04) | 0.72 | - | - |
| **White maternal ethnicity** (white, n=1002; non-white, n=167) | 0.98  (0.58-1.67) | 0.94 | 0.85  (0.45-1.62) | 0.63 | - | - |
| **Antibiotics used in pregnancy**  (yes, n=331; no, n=828) | 1.91  (1.30-2.80) | <0.001 | 2.06  (1.33-3.19) | 0.001 | 2.09  (1.36-3.23) | <0.001 |
| **More than one first degree relative with atopic disease**  (two or more, n=742; one, n=427) | 1.14  (0.77-1.69) | 0.52 | 0.93  (0.58-1.49) | 0.76 | - | - |
| **No other children in household**  (only child, n=486; other children, n=683) | 0.84  (0.57-1.24) | 0.38 | 0.80  (0.50-1.28) | 0.34 | - | - |
| **Exclusive formula feeding from birth to 6 months old**  (formula only, n=151; all other feeding, n=814) | 1.78  (1.09-2.93) | 0.02 | 1.74  (1.03-2.92) | 0.04 | 1.77  (1.06-2.95) | 0.03 |
| **Family decile of English Index of Multiple Deprivation 2015** (n=1146) | 0.97  (0.90-1.04) | 0.35 | 1.00  (0.92-1.08) | 0.95 | - | - |

Logistic regression comparing potential risk factors associated with a positive mention of reaction to milk in the primary care records (n=124) excluding the 19 confirmed milk allergic participants. Participants who did not answer yes to any of the screening questions were assumed not to have a reaction to milk (n=1045). Participants who did not answer any of the screening questions (n=166) and the primary care records that were not received (n=40) were excluded from analysis. Odds ratio (OR) >1 shows a positive association between each variable and a reaction to milk in the records (outcome). Confidence intervals are 95% and p-value <0.05 indicates statistical significance. Unadjusted values indicate an association alone and adjusted values (n=935) consider the association of all variables together on the outcome. †Step 1 of backward stepwise regression including all co-variates ‡Final step of backward stepwise regression

# Table S24. Participant characteristics and primary care record of low-allergy formula prescription (excluding EQ5D)

|  | **Unadjusted** | | **Adjusted** (n=932)† | | **Adjusted** (n=932)‡ | |
| --- | --- | --- | --- | --- | --- | --- |
|  | **OR**  **(95% CI)** | **P-value** | **OR**  **(95% CI)** | **P-value** | **OR**  **(95% CI)** |  |
| **Maternal Age** (n=1164) | 0.97  (0.92-1.01) | 0.10 | 0.97  (0.92-1.03) | 0.33 | - | - |
| **White maternal ethnicity** (white, n=999; non-white, n=165) | 1.19  (0.60-2.35) | 0.63 | 1.21  (0.50-2.96) | 0.67 | - | - |
| **Antibiotics used in pregnancy**  (yes, n=330; no, n=824) | 2.05  (1.30-3.26) | 0.002 | 2.35  (1.38-4.02) | 0.002 | 2.40  (1.41-4.08) | 0.001 |
| **More than one first degree relative with atopic disease**  (two or more, n=738; one, n=426) | 1.24  (0.77-2.01) | 0.38 | 0.91  (0.51-1.62) | 0.74 | - | - |
| **No other children in household**  (only child, n=486; other children, n=678) | 0.96  (0.60-1.51) | 0.85 | 0.85  (0.48-1.53) | 0.59 | - | - |
| **Exclusive formula feeding from birth to 6 months old**  (formula only, n=150; all other feeding, n=811) | 2.32  (1.31-4.13) | 0.004 | 2.08  (1.13-3.82) | 0.02 | 2.21  (1.22-4.01) | 0.009 |
| **Family decile of English Index of Multiple Deprivation 2015** (n=1142) | 0.97  (0.89-1.05) | 0.39 | 1.01  (0.91-1.11) | 0.94 | - | - |

Logistic regression comparing potential risk factors associated with a prescription of low-allergy formula in the primary care records (n=77) excluding the 19 confirmed milk allergic participants. Participants who did not answer yes to any of the screening questions were assumed not to have received a prescription (n=1086). Participants who did not answer any of the screening questions (n=166), the primary care records that were not received (n=40), and the participants with a positive mention of a reaction to milk whose prescription records were missing (n=5) were excluded from analysis. Odds ratio (OR) >1 shows a positive association between each variable and a low-allergy formula prescription (outcome). Confidence intervals are 95% and p-value <0.05 indicates statistical significance. Unadjusted values indicate an association alone and adjusted values (n=932) consider the association of all variables together on the outcome. †Step 1 of backward stepwise regression including all co-variates ‡Final step of backward stepwise regression

# Table S25: Participant characteristics and parent-reported cow’s milk reaction (multiple imputation, 100 imputations).

|  | **Unadjusted** (n=1375) | | **Adjusted** (n=1375) | |
| --- | --- | --- | --- | --- |
|  | **OR**  **(95% CI)** | **P-value** | **OR**  **(95% CI)** | **P-value** |
| **Maternal Age** | 0.98  (0.96-1.02) | 0.34 | 0.99  (0.96-1.02) | 0.54 |
| **White maternal ethnicity** | 1.03  (0.65-1.62) | 0.90 | 1.02  (0.63-1.65) | 0.93 |
| **Antibiotics used in pregnancy** | 1.58  (1.15-2.19) | 0.005 | 1.52  (1.10-2.11) | 0.01 |
| **More than one first degree relative with atopic disease** | 1.03  (0.75-1.43) | 0.85 | 1.02  (0.72-1.44) | 0.93 |
| **No other children in household** | 0.96  (0.70-1.31) | 0.78 | 0.95  (0.68-1.33) | 0.75 |
| **Maternal anxiety/depression on EQ-5D (at baseline)** | 0.96  (0.64-1.43) | 0.84 | 0.85  (0.55-1.30) | 0.46 |
| **Maternal EQ-5D health state at baseline** | 0.99  (0.98-1.00) | 0.18 | 0.99  (0.98-1.01) | 0.19 |
| **Exclusive formula feeding from birth to 6 months old** | 1.38  (0.93-2.06) | 0.11 | 1.34  (0.89-2.01) | 0.16 |
| **Family decile of English Index of Multiple Deprivation 2015** | 0.97  (0.92-1.03) | 0.32 | 0.98  (0.93-1.04) | 0.56 |

Logistic regression comparing participants with a positive response to one of the 3 screening questions excluding the 19 confirmed milk allergic participants (n=195) with the remaining BEEP study cohort (n=1180). Multiple imputation (100 imputations) was used for missing data within the exposure and outcome variables. Odds ratio (OR) >1 shows a positive association between each variable and parental reported reaction to milk or low-allergy formula prescription (outcome). Confidence intervals are 95% and p-value <0.05 indicates statistical significance. Unadjusted values indicate an association alone and adjusted values consider the association of all variables together on the outcome.

#

# Table S26: Participant characteristics and primary care record of cow’s milk hypersensitivity (multiple imputation, 100 imputations).

|  | **Unadjusted** (n=1375) | | **Adjusted** (n=1375) | |
| --- | --- | --- | --- | --- |
|  | **OR**  **(95% CI)** | **P-value** | **OR**  **(95% CI)** | **P-value** |
| **Maternal Age** | 0.99  (0.95-1.02) | 0.43 | 0.99  (0.95-1.03) | 0.62 |
| **White maternal ethnicity** | 0.96  (0.57-1.61) | 0.87 | 0.95  (0.55-1.62) | 0.84 |
| **Antibiotics used in pregnancy** | 1.90  (1.31-2.77) | <0.001 | 1.83  (1.25-2.68) | <0.002 |
| **More than one first degree relative with atopic disease** | 1.14  (0.77-1.69) | 0.52 | 1.10  (0.72-1.69) | 0.66 |
| **No other children in household** | 0.84  (0.58-1.23) | 0.38 | 0.86  (0.57-1.30) | 0.48 |
| **Maternal anxiety/depression on EQ-5D (at baseline)** | 1.03  (0.63-1.67) | 0.91 | 0.92  (0.55-1.53) | 0.74 |
| **Maternal EQ-5D health state at baseline** | 0.99  (0.98-1.00) | 0.26 | 0.99  (0.98-1.01) | 0.34 |
| **Exclusive formula feeding from birth to 6 months old** | 1.52  (0.95-2.44) | 0.08 | 1.47  (0.91-2.38) | 0.12 |
| **Family decile of English Index of Multiple Deprivation 2015** | 0.97  (0.90-1.04) | 0.33 | 0.98  (0.91-1.06) | 0.63 |

Logistic regression comparing potential risk factors associated with a positive mention of reaction to milk in the primary care records (n=124) excluding the 19 confirmed milk allergic participants. Multiple imputation (100 imputations) was used for missing data within the exposure and outcome variables. Odds ratio (OR) >1 shows a positive association between each variable and a reaction to milk in the records (outcome). Confidence intervals are 95% and p-value <0.05 indicates statistical significance. Unadjusted values indicate an association alone and adjusted values consider the association of all variables together on the outcome.

# Table S27: Participant characteristics and primary care record of low-allergy formula prescription (multiple imputation, 100 imputations).

|  | **Unadjusted** (n=1375) | | **Adjusted** (n=1375) | |
| --- | --- | --- | --- | --- |
|  | **OR**  **(95% CI)** | **P-value** | **OR**  **(95% CI)** | **P-value** |
| **Maternal Age** | 0.96  (0.92-1.01) | 0.11 | 0.97  (0.93-1.02) | 0.27 |
| **White maternal ethnicity** | 1.14  (0.59-2.20) | 0.71 | 1.06  (0.53-2.11) | 0.88 |
| **Antibiotics used in pregnancy** | 2.00  (1.27-3.16) | 0.003 | 1.91  (1.20-3.05) | 0.007 |
| **More than one first degree relative with atopic disease** | 1.21  (0.74-1.99) | 0.45 | 1.22  (0.72-2.07) | 0.46 |
| **No other children in household** | 0.94  (0.60-1.49) | 0.80 | 0.97  (0.59-1.60) | 0.90 |
| **Maternal anxiety/depression on EQ-5D (at baseline)** | 1.00  (0.57-1.78) | 0.99 | 0.91  (0.50-1.64) | 0.75 |
| **Maternal EQ-5D health state at baseline** | 0.99  (0.98-1.01) | 0.53 | 1.00  (0.98-1.02) | 0.64 |
| **Exclusive formula feeding from birth to 6 months old** | 1.84  (1.07-3.15) | 0.03 | 1.73  (0.98-3.02) | 0.06 |
| **Family decile of English Index of Multiple Deprivation 2015** | 0.96  (0.89-1.05) | 0.38 | 0.99  (0.90-1.08) | 0.75 |

Logistic regression comparing potential risk factors associated with a prescription of low-allergy formula in the primary care records (n=77) excluding the 19 confirmed milk allergic participants. Participants who did not answer yes to any of the screening questions were assumed not to have received a prescription. Multiple imputation (100 imputations) was used for missing data within the exposure and outcome variables. Odds ratio (OR) >1 shows a positive association between each variable and a low-allergy formula prescription (outcome). Confidence intervals are 95% and p-value <0.05 indicates statistical significance. Unadjusted values indicate an association alone and adjusted values consider the association of all variables together on the outcome.

#

# Table S28. Clustering of BEEP participants in primary care practices

|  | **All BEEP participants†**  **Practices, n=640** | **BEEP participants excluding confirmed CMA†**  **Practices, n=636** |
| --- | --- | --- |
| **Practices with 1 participant** | 352 (55%) | 350 (55%) |
| **Practices with >1 participant** | 288 (45%) | 286 (45%) |

†No practice code was available for 14 participants in the total cohort and 13 participants without confirmed CMA. Variance between practices or within practices could not be assessed due to the presence of singleton clusters.

#

# Table S29: Practice-level variables comparing participants with and without parent-reported cow’s milk hypersensitivity

|  | **Unadjusted** | | **Adjusted** (n=1193)† | | **Adjusted** (n=1193)‡ | |
| --- | --- | --- | --- | --- | --- | --- |
|  | **OR**  **(95% CI)** | **P-value** | **OR**  **(95% CI)** | **P-value** | **OR**  **(95% CI)** | **P-value** |
| **Practice volume of low-allergy formula prescription (litres per infant aged 0–1-year practice population)** (n=1194) | 1.03  (1.01-1.05) | <0.001 | 1.03  (1.01-1.05) | 0.002 | 1.03  (1.02-1.05) | <0.001 |
| **Practice antibiotic prescriptions (Items per 1000 total practice population)** (n=1194) | 0.999  (0.998-1.00) | 0.17 | 0.995  (0.99-1.00) | 0.05 | 0.996  (0.992-1.00) | 0.09 |
| **Practice Decile of English Index of Multiple Deprivation (IMD) 2019**  (n=1194) | 1.00  (0.94-1.05) | 0.88 | 0.97  (0.91-1.03) | 0.33 | - | - |
| **Practice anti-reflux prescriptions (Items per infant aged 0–1-year practice population)**  (n=1194) | 1.02  (1.00-1.05) | 0.11 | 1.03  (1.00-1.05) | 0.06 | - | - |
| **Practice Gaviscon® Infant prescriptions (Quantity per infant aged 0–1-year practice population)**  (n=1194) | 1.00  (1.00-1.01) | 0.46 | 1.00  (0.99-1.01) | 0.85 | - | - |
| **Practice AAI prescriptions (Items per 1000 aged 0-5 years practice population)**  (n=1194) | 1.00  (1.00-1.01) | 0.27 | 1.00  (1.00-1.01) | 0.67 | - | - |
| **CCG CMA guidelines highlights reproducibility or specificity as criteria for CMA diagnosis (**n=1197) | 1.06  (0.90-1.26) | 0.50 | 1.59  (0.96-2.63) | 0.07 | - | - |
| **Antibiotic Items/STARPU (Average of 4 quarters for 2014)§** (n=1195) | 0.12  (0.01-1.46 | 0.10 | 0.97  (0.01-1.58) | 0.10 | 0.10  (0.01-1.30) | 0.08 |

Logistic regression comparing practice-level variables of those participants with a positive response to one of the 3 screening questions excluding the 19 confirmed milk allergic participants (n=195) with the remaining BEEP study cohort who answered the screening questions (n=1014). BEEP participants who did not answer the screening questions were excluded from analysis (n=166). No data was available for 15 GP practices. Odds ratio (OR) >1 shows a positive association between each variable and parental reported reaction to milk or low-allergy formula prescription (outcome). Confidence intervals are 95% and p-value <0.05 indicates statistical significance. Unadjusted values indicate an association alone and adjusted values (n=1193) consider the association of all variables together on the outcome. †Step 1 of backward stepwise regression including all co-variates ‡Final step of backward stepwise regression. Practice-level variables were assessed for the year prior to the BEEP study (2014) except IMD which was recorded for 2019 as this was the year used in the BEEP clinical trial for participant IMD. §Antibiotic Items/STARPU was substituted for practice antibiotic prescription items as a predictor variable to adjust for gender and sex effect on antibiotic prescribing in the practice.

# Table S30: Practice-level variables comparing participants with and without a primary care record of cow’s milk hypersensitivity

|  | **Unadjusted** | | **Adjusted** (n=1153)† | | **Adjusted** (n=1153)‡ | |
| --- | --- | --- | --- | --- | --- | --- |
|  | **OR**  **(95% CI)** | **P-value** | **OR**  **(95% CI)** | **P-value** | **OR**  **(95% CI)** | **P-value** |
| **Practice volume of low-allergy formula prescription (litres per infant aged 0–1-year practice population)** (n=1154) | 1.04  (1.02-1.06) | <0.001 | 1.04  (1,02-1.06) | <0.001 | 1.04  (1.02-1.06) | <0.001 |
| **Practice antibiotic prescriptions (Items per 1000 total practice population)** (n=1154) | 0.999  (0.998-1.00) | 0.07 | 0.998  (0.997-1.00) | 0.02 | 0.999  (0.997-1.00) | 0.03 |
| **Practice Decile of English Index of Multiple Deprivation (IMD) 2019**  (n=1154) | 0.99  (0.93-1.06) | 0.79 | 0.96  (0.89-1.04) | 0.3 | - | - |
| **Practice anti-reflux prescriptions (Items per infant aged 0–1-year practice population)**  (n=1154) | 1.02  (0.99-1.05) | 0.19 | 1.03  (1.00-1.06) | 0.09 | - | - |
| **Practice Gaviscon® Infant prescriptions (Quantity per infant aged 0–1-year practice population)**  (n=1154) | 1.00  (1.00-1.01) | 0.37 | 1.00  (0.99-.01) | 0.60 | - | - |
| **Practice AAI prescriptions (Items per 1000 aged 0-5 years practice population)**  (n=1154) | 1.00  (1.00-1.01) | 0.18 | 1.00  (1.00-1.01 | 0.64 | - | - |
| **CCG CMA guidelines highlights reproducibility or specificity as criteria for CMA diagnosis (**n=1157) | 1.87  (1.05-3.34) | 0.33 | 1.57  (0.86-2.86) | 0.14 | - | - |
| **Antibiotic Items/STAR-PU (Average of 4 quarters for 2014)§** (n=1155) | 0.04  (0.002-0.91) | 0.04 | 0.16  (0.00-0.52) | 0.02 | 0.03  (0.00-0.72) | 0.03 |

Logistic regression comparing potential risk factors associated with a positive mention of reaction to milk in the primary care records (n=124) excluding the 19 confirmed milk allergic participants. Participants who did not answer yes to any of the screening questions were assumed not to have a reaction to milk (n=1045). Participants who did not answer any of the screening questions (n=166) and the primary care records that were not received (n=40) were excluded from analysis. No data was available for 15 GP practices. Odds ratio (OR) >1 shows a positive association between each variable and a reaction to milk in the records (outcome). Confidence intervals are 95% and p-value <0.05 indicates statistical significance. Unadjusted values indicate an association alone and adjusted values (n=1153) consider the association of all variables together on the outcome. †Step 1 of backward stepwise regression including all co-variates ‡Final step of backward stepwise regression. Practice-level variables were assessed for the year prior to the BEEP study (2014) except IMD which was recorded for 2019 as this was the year used in the BEEP clinical trial for participant IMD. §Antibiotic Items/STARPU was substituted for practice antibiotic prescription items as a predictor variable to adjust for gender and sex effect on antibiotic prescribing in the practice.

# Table S31: Practice-level variables comparing participants with and without a primary care record of low-allergy formula prescription

|  | **Unadjusted** | | **Adjusted** (n=1148)† | | **Adjusted** (n=1148)‡ | |
| --- | --- | --- | --- | --- | --- | --- |
|  | **OR**  **(95% CI)** | **P-value** | **OR**  **(95% CI)** | **P-value** | **OR**  **(95% CI)** | **P-value** |
| **Practice volume of low-allergy formula prescription (litres per infant aged 0–1-year practice population)** (n=1149) | 1.04  (1.02-1.07) | <0.001 | 1.04  (1.02-1.07) | 0.001 | 1.04  (1.02-1.07) | <0.001 |
| **Practice antibiotic prescriptions (Items per 1000 total practice population)** (n=1149) | 0.999  (0.997-1.00) | 0.07 | 0.998  (0.996-1.00) | 0.02 | 0.998  (0.997-1.00) | 0.03 |
| **Practice Decile of English Index of Multiple Deprivation (IMD) 2019**  (n=1149) | 0.98  (0.90-1.06) | 0.60 | 0.96  (0.88-1.05) | 0.36 | - | - |
| **Practice anti-reflux prescriptions (Items per infant aged 0–1-year practice population)**  (n=1149) | 1.01  (0.98-1.05) | 0.47 | 1.02  (0.99-1.06) | 0.23 | - | - |
| **Practice Gaviscon® Infant prescriptions (Quantity per infant aged 0–1-year practice population)**  (n=1149) | 1.00  (0.99-1.01) | 0.65 | 1.00  (0.99-1.01) | 0.98 | - | - |
| **Practice AAI prescriptions (Items per 1000 aged 0-5 years practice population)**  (n=1149) | 1.00  (1.00-1.01) | 0.68 | 1.00  (0.99-1.01) | 0.69 | - | - |
| **CCG CMA guidelines highlights reproducibility or specificity as criteria for CMA diagnosis (**n=1152) | 1.97  (1.00-3.87) | 0.05 | 1.64  (0.81-3.32) | 0.17 | - | - |
| **Antibiotic Items/STAR-PU (Average of 4 quarters for 2014)§** (n=1150) | 0.02  (0.00-0.94) | 0.05 | 0.01  (0.00-0.49) | 0.02 | 0.02  (0.00-0.73) | 0.03 |

Logistic regression comparing potential risk factors associated with a prescription of low-allergy formula in the primary care records (n=77) excluding the 19 confirmed milk allergic participants. Participants who did not answer yes to any of the screening questions were assumed not to have received a prescription (n=1086). Participants who did not answer any of the screening questions (n=166), the primary care records that were not received (n=40), and the participants with a positive mention of a reaction to milk whose prescription records were missing (n=5) were excluded from analysis. No data was available for 15 GP practices. Odds ratio (OR) >1 shows a positive association between each variable and a low-allergy formula prescription (outcome). Confidence intervals are 95% and p-value <0.05 indicates statistical significance. Unadjusted values indicate an association alone and adjusted values (n=1148) consider the association of all variables together on the outcome. †Step 1 of backward stepwise regression including all co-variates ‡Final step of backward stepwise regression. Practice-level variables were assessed for the year prior to the BEEP study (2014) except IMD which was recorded for 2019 as this was the year used in the BEEP clinical trial for participant IMD. §Antibiotic Items/STARPU was substituted for practice antibiotic prescription items as a predictor variable to adjust for gender and sex effect on antibiotic prescribing in the practice.

# Table S32: Combined participant and practice-level variables comparing participants with and without parent-reported cow’s milk hypersensitivity

|  | **Adjusted** (n=529)† | | **Adjusted** (n=529)‡ | |
| --- | --- | --- | --- | --- |
|  | **OR**  **(95% CI)** | **P-value** | **OR**  **(95% CI)** | **P-value** |
| **Maternal Age** (n=1209) | 0.96  (0.91-1.01) | 0.12 | - | - |
| **White maternal ethnicity** (white, n=1037; non-white, n=172) | 0.56  (0.23-1.35) | 0.20 | - | - |
| **Antibiotics used in pregnancy**  (yes, n=345; no, n=854) | 1.95  (1.17-3.27) | 0.01 | 1.97  (1.19-3.26) | 0.008 |
| **More than one first degree relative with atopic disease**  (two or more, n=767; one, n=442) | 1.11  (0.64-1.92) | 0.72 | - | - |
| **No other children in household**  (only child, n=506; other children, n=703) | 1.00  (0.58-1.71) | 0.99 | - | - |
| **Maternal anxiety/depression on EQ-5D (at baseline)**  (any anxiety, n=188; no anxiety, n=709) | 0.64  (0.32-1.29) | 0.21 | - | - |
| **Maternal EQ-5D health state at baseline** (n=891) | 0.98  (0.96-1.00) | 0.03 | 0.98  (0.96-1.00) | 0.06 |
| **Exclusive formula feeding from birth to 6 months old**  (formula only, n=157; all other feeding, n=837) | 1.80  (0.96-3.37) | 0.07 | 1.73  (0.94-3.18) | 0.08 |
| **Practice volume of low-allergy formula prescription (litres per infant aged 0–1-year practice population)** (n=1149) | 1.03  (0.96-1.05) | 0.11 | 1.03  (1.00-1.05) | 0.05 |
| **Practice antibiotic prescriptions (Items per 1000 total practice population)** (n=1149) | 1.00  (0.99-1.00) | 0.78 | - | - |
| **Practice Decile of English Index of Multiple Deprivation (IMD) 2019**  (n=1149) | 1.04  (0.95-1.15) | 0.41 | - | - |
| **Practice anti-reflux prescriptions (Items per infant aged 0–1-year practice population)**  (n=1149) | 1.00  (0.95-1.05) | 0.99 | - | - |
| **Practice Gaviscon® Infant prescriptions (Quantity per infant aged 0–1-year practice population)**  (n=1149) | 1.00  (0.99-1.01) | 0.81 | - | - |
| **Practice AAI prescriptions (Items per 1000 aged 0-5 years practice population)**  (n=1149) | 1.00  (0.99-1.01) | 0.94 | - | - |
| **CCG CMA guidelines highlights reproducibility or specificity as criteria for CMA diagnosis (**n=1152) | 1.91  (1.01-3.63) | 0.05 | 1.86  (0.99-3.46) | 0.05 |

Logistic regression comparing combined participant and practice-level variables of those participants with a positive response to one of the 3 screening questions excluding the 19 confirmed milk allergic participants (n=195) with the remaining BEEP study cohort who answered the screening questions (n=1014). BEEP participants who did not answer the screening questions were excluded from analysis (n=166). Odds ratio (OR) >1 shows a positive association between each variable and parental reported reaction to milk or low-allergy formula prescription (outcome). Confidence intervals are 95% and p-value <0.05 indicates statistical significance. Unadjusted values indicate an association alone and adjusted values (n=1193) consider the association of all variables together on the outcome. †Step 1 of backward stepwise regression including all co-variates ‡Final step of backward stepwise regression. Practice-level variables were assessed for the year prior to the BEEP study (2014) except IMD which was recorded for 2019 as this was the year used in the BEEP clinical trial for participant IMD.

# Table S33: Combined participant and practice-level variables comparing participants with and without a primary care record of cow’s milk hypersensitivity

|  | **Adjusted** (n=515)† | | | **Adjusted** (n=515)† | |
| --- | --- | --- | --- | --- | --- |
|  | **OR**  **(95% CI)** | **P-value** | | **OR**  **(95% CI)** | **P-value** |
| **Maternal Age** (n=1209) | 0.97  (0.91-1.03) | | 0.31 | - | - |
| **White maternal ethnicity** (white, n=1037; non-white, n=172) | 0.36  (0.14-0.95) | | 0.04 | 0.43  (0.17-1.06) | 0.07 |
| **Antibiotics used in pregnancy**  (yes, n=345; no, n=854) | 2.09  (1.12-3.88) | | 0.02 | 2.17  (1.19-3.97) | 0.01 |
| **More than one first degree relative with atopic disease**  (two or more, n=767; one, n=442) | 1.33  (0.67-2.64) | | 0.42 | - | - |
| **No other children in household**  (only child, n=506; other children, n=703) | 0.92  (0.48-1.78) | | 0.80 | - | - |
| **Maternal anxiety/depression on EQ-5D (at baseline)**  (any anxiety, n=188; no anxiety, n=709) | 0.91  (0.41-2.02) | | 0.81 | - | - |
| **Maternal EQ-5D health state at baseline** (n=891) | 0.98  (0.96-1.01) | | 0.10 | 0.98  (0.96-1.00) | 0.07 |
| **Exclusive formula feeding from birth to 6 months old**  (formula only, n=157; all other feeding, n=837) | 2.52  (1.23-5.19) | | 0.01 | 2.39  (1.19-4.78) | 0.01 |
| **Practice volume of low-allergy formula prescription (litres per infant aged 0–1-year practice population)** (n=1154) | 1.03  (1.00-1.06) | 0.08 | | 1.04  (1.01-1.07) | 0.007 |
| **Practice antibiotic prescriptions (Items per 1000 total practice population)** (n=1154) | 0.99  (0.99-1.00) | 0.59 | | - | - |
| **Practice Decile of English Index of Multiple Deprivation (IMD) 2019**  (n=1154) | 1.06  (0.94-1.19) | 0.38 | | - | - |
| **Practice anti-reflux prescriptions (Items per infant aged 0–1-year practice population)**  (n=1154) | 1.00  (0.94-1.07) | 0.94 | | - | - |
| **Practice Gaviscon® Infant prescriptions (Quantity per infant aged 0–1-year practice population)**  (n=1154) | 1.00  (0.99-1.02) | 0.63 | | - | - |
| **Practice AAI prescriptions (Items per 1000 aged 0-5 years practice population)**  (n=1154) | 1.01  (1.00-1.01) | 0.32 | | - | - |
| **CCG CMA guidelines highlights reproducibility or specificity as criteria for CMA diagnosis (**n=1157) | 1.60  (0.73-3.53) | 0.24 | | - | - |

Logistic regression comparing combined participant and practice-level variables associated with a positive mention of reaction to milk in the primary care records (n=124) excluding the 19 confirmed milk allergic participants. Participants who did not answer yes to any of the screening questions were assumed not to have a reaction to milk (n=1045). Participants who did not answer any of the screening questions (n=166) and the primary care records that were not received (n=40) were excluded from analysis. Odds ratio (OR) >1 shows a positive association between each variable and a reaction to milk in the records (outcome). Confidence intervals are 95% and p-value <0.05 indicates statistical significance. Unadjusted values indicate an association alone and adjusted values (n=1153) consider the association of all variables together on the outcome. †Step 1 of backward stepwise regression including all co-variates ‡Final step of backward stepwise regression. Practice-level variables were assessed for the year prior to the BEEP study (2014) except IMD which was recorded for 2019 as this was the year used in the BEEP clinical trial for participant IMD.

# Table S34: Combined participant and practice-level variables comparing participants with and without a primary care record of low-allergy formula prescription

|  | **Adjusted** (n=514)† | | **Adjusted** (n=514)‡ | |
| --- | --- | --- | --- | --- |
|  | **OR**  **(95% CI)** | **P-value** | **OR**  **(95% CI)** | **P-value** |
| **Maternal Age** (n=1209) | 0.95  (0.88-1.02) | 0.15 | - | - |
| **White maternal ethnicity** (white, n=1037; non-white, n=172) | 0.41  (0.13-1.22) | 0.11 | - | - |
| **Antibiotics used in pregnancy**  (yes, n=345; no, n=854) | 2.10  (1.02-4.29) | 0.04 | 2.42  (1.22-4.82) | 0.01 |
| **More than one first degree relative with atopic disease**  (two or more, n=767; one, n=442) | 1.50  (0.67-3.34) | 0.32 | - | - |
| **No other children in household**  (only child, n=506; other children, n=703) | 1.15  (0.54-2.45) | 0.71 | - | - |
| **Maternal anxiety/depression on EQ-5D (at baseline)**  (any anxiety, n=188; no anxiety, n=709) | 0.74  (0.28-1.96) | 0.54 | - | - |
| **Maternal EQ-5D health state at baseline** (n=891) | 0.98  (0.95-1.01) | 0.12 | - | - |
| **Exclusive formula feeding from birth to 6 months old**  (formula only, n=157; all other feeding, n=837) | 3.16  (1.42-7.02) | 0.005 | 2.87  (1.35-6.10) | 0.006 |
| **Practice volume of low-allergy formula prescription (litres per infant aged 0–1-year practice population)** (n=1149) | 1.04  (1.02-1.07) | 0.001 | 1.04  (1.02-1.07) | <0.001 |
| **Practice antibiotic prescriptions (Items per 1000 total practice population)** (n=1149) | 0.998  (0.996-1.00) | 0.02 | - | - |
| **Practice Decile of English Index of Multiple Deprivation (IMD) 2019**  (n=1149) | 0.96  (0.88-1.05) | 0.36 | - | - |
| **Practice anti-reflux prescriptions (Items per infant aged 0–1-year practice population)**  (n=1149) | 1.02  (0.99-1.06) | 0.23 | - | - |
| **Practice Gaviscon® Infant prescriptions (Quantity per infant aged 0–1-year practice population)**  (n=1149) | 1.00  (0.99-1.01) | 0.98 | - | - |
| **Practice AAI prescriptions (Items per 1000 aged 0-5 years practice population)**  (n=1149) | 1.00  (0.99-1.01) | 0.69 | - | - |
| **CCG CMA guidelines highlights reproducibility or specificity as criteria for CMA diagnosis (**n=1152) | 1.64  (0.81-3.32) | 0.17 | - | - |

Logistic regression comparing potential risk factors associated with a prescription of low-allergy formula in the primary care records (n=77) excluding the 19 confirmed milk allergic participants. Participants who did not answer yes to any of the screening questions were assumed not to have received a prescription (n=1086). Participants who did not answer any of the screening questions (n=166), the primary care records that were not received (n=40), and the participants with a positive mention of a reaction to milk whose prescription records were missing (n=5) were excluded from analysis. Odds ratio (OR) >1 shows a positive association between each variable and a low-allergy formula prescription (outcome). Confidence intervals are 95% and p-value <0.05 indicates statistical significance. Unadjusted values indicate an association alone and adjusted values (n=1148) consider the association of all variables together on the outcome. †Step 1 of backward stepwise regression including all co-variates ‡Final step of backward stepwise regression. Practice-level variables were assessed for the year prior to the BEEP study (2014) except IMD which was recorded for 2019 as this was the year used in the BEEP clinical trial for participant IMD.

# Table S35: Combined participant and practice-level variables comparing participants with and without parent-reported cow’s milk reaction (multiple imputation, 100 imputations)

|  | **Adjusted** (n=1356) | |
| --- | --- | --- |
|  | **OR**  **(95% CI)** | **P-value** |
| **Maternal Age** | 0.98  (0.95-1.01) | 0.26 |
| **White maternal ethnicity** | 0.97  (0.59-1.58) | 0.90 |
| **Antibiotics used in pregnancy** | 1.53  (1.10-2.14) | 0.01 |
| **More than one first degree relative with atopic disease** | 1.02  (0.72-1.47) | 0.90 |
| **No other children in household** | 0.89  (0.63-1.27) | 0.53 |
| **Maternal anxiety/depression on EQ-5D (at baseline)** | 0.77  (0.49-1.20) | 0.24 |
| **Maternal EQ-5D health state at baseline** | 0.99  (0.98-1.00) | 0.15 |
| **Exclusive formula feeding from birth to 6 months old** | 1.40  (0.93-2.11) | 0.11 |
| **Practice volume of low-allergy formula prescription (litres per infant aged 0-1 year practice population** | 1.03  (1.01-1.04) | 0.006 |
| **Practice antibiotic prescriptions (Items per 1000 total practice population)** | 1.00  (0.99-1.00) | 0.08 |
| **Practice Decile of English Index of Multiple Deprivation (IMD) 2019** | 0.97  (0.92-1.04) | 0.41 |
| **Practice anti-reflux prescriptions (Items per infant aged 0-1 year practice population)** | 1.03  (1.00-1.05) | 0.08 |
| **Practice Gaviscon® Infant prescriptions (Quantity per infant aged 0-1 year practice population)** | 1.00  (0.99-1.01) | 0.95 |
| **Practice AAI prescriptions (Items per 1000 aged 0-5 years practice population)** | 1.00  (1.00-1.01) | 0.58 |
| **CCG CMA guidelines highlights reproducibility or specificity as criteria for CMA diagnosis** | 1.45  (0.87-2.41) | 0.15 |

Logistic regression comparing combined participant and practice-level variables of those participants with a positive response to one of the 3 screening questions excluding the 19 confirmed milk allergic participants (n=195) with the remaining BEEP study cohort who answered the screening questions (n=1014). Odds ratio (OR) >1 shows a positive association between each variable and parental reported reaction to milk or low-allergy formula prescription (outcome). Confidence intervals are 95% and p-value <0.05 indicates statistical significance. Adjusted values (n=1356) consider the association of all variables together on the outcome. Practice-level variables were assessed for the year prior to the BEEP study (2014) except IMD which was recorded for 2019 as this was the year used in the BEEP clinical trial for participant IMD. Multiple imputation (100 imputations) was used for missing participants data only within the exposure and outcome variables. Practice determinants were missing for 19 participants.

# Table S36: Combined participant and practice-level variables comparing participants with and without a primary care record of cow’s milk hypersensitivity (multiple imputation, 100 imputations)

|  | **Adjusted** (n=1356) | | |
| --- | --- | --- | --- |
|  | **OR**  **(95% CI)** | **P-value** | |
| **Maternal Age** | 0.98  (0.94-1.02) | | 0.31 |
| **White maternal ethnicity** | 0.90  (0.51-1.59) | | 0.72 |
| **Antibiotics used in pregnancy** | 1.82  (1.23-2.69) | | 0.003 |
| **More than one first degree relative with atopic disease** | 1.10  (0.70-1.72) | | 0.68 |
| **No other children in household** | 0.78  (0.50-1.20) | | 0.25 |
| **Maternal anxiety/depression on EQ-5D (at baseline)** | 0.79  (0.46-1.38) | | 0.41 |
| **Maternal EQ-5D health state at baseline** | 0.99  (0.98-1.00) | | 0.30 |
| **Exclusive formula feeding from birth to 6 months old** | 1.56  (0.95-2.58) | | 0.08 |
| **Practice volume of low-allergy formula prescription (litres per infant aged 0-1 year practice population)** | 1.03  (1.00-1.05) | 0.007 | |
| **Practice antibiotic prescriptions (Items per 1000 total practice population)** | 0.99  (0.99-1.00) | 0.06 | |
| **Practice Decile of English Index of Multiple Deprivation (IMD) 2019** | 0.97  (0.90-1.04) | 0.37 | |
| **Practice anti-reflux prescriptions (Items per infant aged 0-1 year practice population)** | 1.02  (0.99-1.05) | 0.14 | |
| **Practice Gaviscon® Infant prescriptions (Quantity per infant aged 0-1 year practice population)** | 1.00  (0.99-1.01) | 0.65 | |
| **Practice AAI prescriptions (Items per 1000 aged 0-5 years practice population)** | 1.00  (1.00-1.01) | 0.63 | |
| **CCG CMA guidelines highlights reproducibility or specificity as criteria for CMA diagnosis** | 1.42  (0.77-2.60) | 0.26 | |

Logistic regression comparing combined participant and practice-level variables associated with a positive mention of reaction to milk in the primary care records (n=124) excluding the 19 confirmed milk allergic participants. Participants who did not answer yes to any of the screening questions were assumed not to have a reaction to milk (n=1045). Odds ratio (OR) >1 shows a positive association between each variable and a reaction to milk in the records (outcome). Confidence intervals are 95% and p-value <0.05 indicates statistical significance. Adjusted values (n=1356) consider the association of all variables together on the outcome. Practice-level variables were assessed for the year prior to the BEEP study (2014) except IMD which was recorded for 2019 as this was the year used in the BEEP clinical trial for participant IMD. Multiple imputation (100 imputations) was used for missing participants data only within the exposure and outcome variables. Practice determinants were missing for 19 participants.

#

# Table S37: Combined participant and practice-level variables comparing participants with and without a primary care record of low-allergy formula prescription (multiple imputation, 100 imputations)

|  | **Adjusted** (n=1356) | |
| --- | --- | --- |
|  | **OR**  **(95% CI)** | **P-value** |
| **Maternal Age** | 0.96  (0.92-1.01) | 0.13 |
| **White maternal ethnicity** | 1.04  (0.50-2.15) | 0.92 |
| **Antibiotics used in pregnancy** | 1.85  (1.15-3.00) | 0.01 |
| **More than one first degree relative with atopic disease** | 1.23  (0.71-2.13) | 0.46 |
| **No other children in household** | 0.88  (0.52-1.48) | 0.63 |
| **Maternal anxiety/depression on EQ-5D (at baseline)** | 0.81  (0.43-1.51) | 0.50 |
| **Maternal EQ-5D health state at baseline** | 1.00  (0.98-1.01) | 0.59 |
| **Exclusive formula feeding from birth to 6 months old** | 1.86  (1.04-3.31) | 0.04 |
| **Practice volume of low-allergy formula prescription (litres per infant aged 0-1 year practice population)** | 1.03  (1.01-1.06) | 0.01 |
| **Practice antibiotic prescriptions (Items per 1000 total practice population)** | 0.99  (0.99-1.00) | 0.06 |
| **Practice Decile of English Index of Multiple Deprivation (IMD) 2019** | 0.97  (0.88-1.06) | 0.46 |
| **Practice anti-reflux prescriptions (Items per infant aged 0-1 year practice population)** | 1.02  (0.98-1.05) | 0.37 |
| **Practice Gaviscon® Infant prescriptions (Quantity per infant aged 0-1 year practice population)** | 1.00  (0.99-1.01) | 0.95 |
| **Practice AAI prescriptions (Items per 1000 aged 0-5 years practice population)** | 1.00  (0.99-1.01) | 0.92 |
| **CCG CMA guidelines highlights reproducibility or specificity as criteria for CMA diagnosis** | 1.45  (0.72-2.93) | 0.30 |

Logistic regression comparing potential risk factors associated with a prescription of low-allergy formula in the primary care records (n=77) excluding the 19 confirmed milk allergic participants. Participants who did not answer yes to any of the screening questions were assumed not to have received a prescription (n=1086). Odds ratio (OR) >1 shows a positive association between each variable and a low-allergy formula prescription (outcome). Confidence intervals are 95% and p-value <0.05 indicates statistical significance. Adjusted values (n=1356) consider the association of all variables together on the outcome. Practice-level variables were assessed for the year prior to the BEEP study (2014) except IMD which was recorded for 2019 as this was the year used in the BEEP clinical trial for participant IMD. Multiple imputation (100 imputations) was used for missing participants data only within the exposure and outcome variables. Practice determinants were missing for 19 participants.

#

# Table S38: BEEP participant characteristics compared to general population England 2014

|  | **Livebirths England 2014**  **n=661,496** | **All BEEP participants**  **n=1394** | **BEEP excluding infants with confirmed CMA**  **n=1375** |
| --- | --- | --- | --- |
| **Maternal age at randomisation / birth** | 30.2 | 31.65 (28, 35) | 31.62 (28, 35) |
| **White maternal ethnicity** | 479,567 (72.5%)† | 1190 (85.4%) | 1177 (85.6%) |
| **Family decile of English Index of Multiple Deprivation 2015 (based on Integer 1-10, where 1 is the most deprived)** | 4 (2, 6)‡ | 5.85 (3, 8.75) | 5.85 (3, 9) |
| **Exclusive formula feeding from birth** | 24%§ | 165 (11.8%) | 164 (11.9%) |
| **FLG genotyping¶**  **No mutation**  **One FLG null mutation**  **Two FLG null mutations** | 677/789 (86%)  103/789 (13%)  9/789 (1%) | 691/816 (84.7%)  122/816 (15%)  3/816 (0.4%) | 683/805 (84.8%)  119/805 (14.8%)  3/805 (0.4%) |

 †Reported as ethnicity of infant defined by the mother. (11) ‡ NHS Maternity statistics 2013-15 (12) §Source: Table 1: Initiation of breastfeeding, England Trend. 2013/2014. (13) **¶**Filaggrin (FLG) null mutation rate was based on a population-based study in England as comparator. (14) BEEP study participants included in analysis for FLG genotyping (n=816).

# Figure S1. Overlap between categories of CMA overdiagnosis


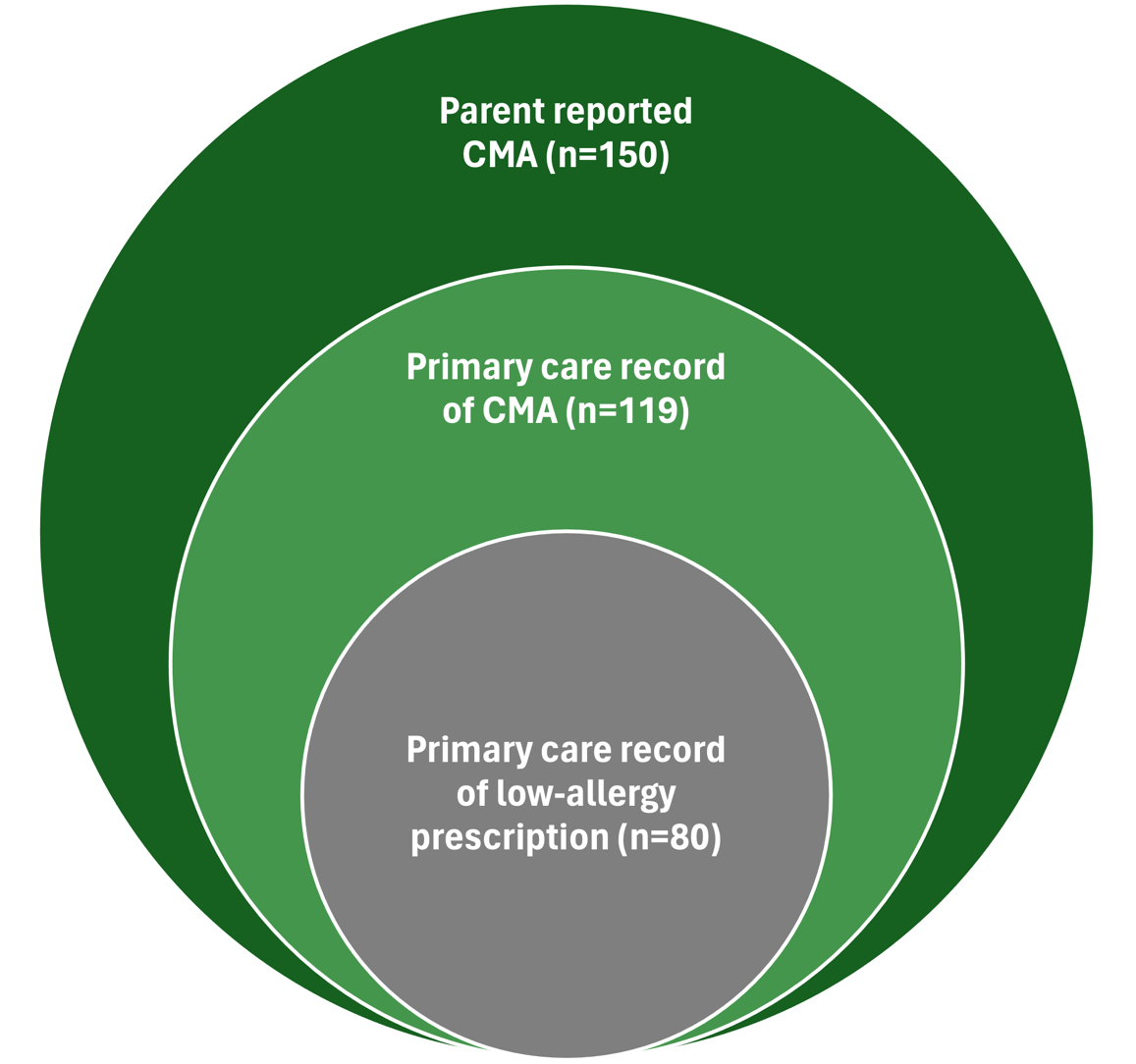


Diagram showing the overlap between parent-reported CMA, primary care record of documented CMA and primary care record of documented low-allergy formula prescription. One further child had a low-allergy formula prescribed without a primary care record of CMA diagnosis (diagnosis was protein-losing enteropathy). Data is for participants with complete data for all 3 parameters, but excluding those who had proven CMA.

# Figure S2. Timing of initial symptoms and diagnosis of possible cow’s milk hypersensitivity

**(A)** **(B)**


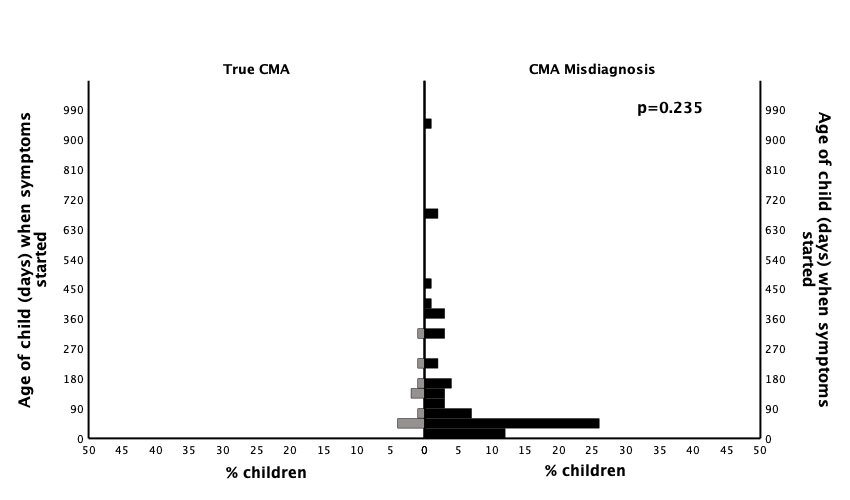

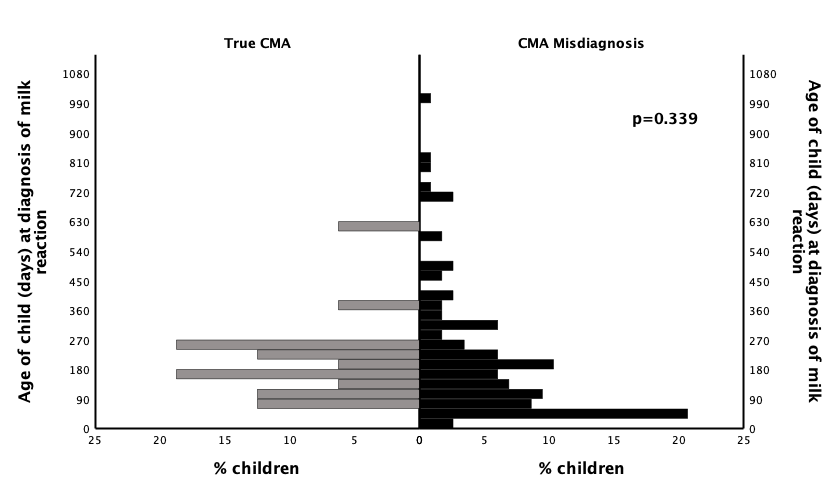


**(C)**


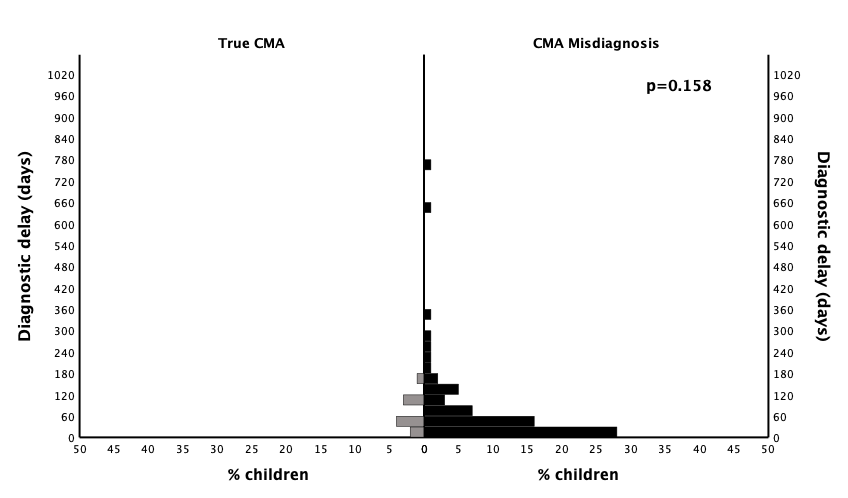


Histograms showing (A) age of child (days) when symptoms relevant for diagnosis first started (CMA overdiagnosis, n=68 ; confirmed CMA, n=10 ) and (B) age of child (days) at diagnosis of reaction to cow’s milk (CMA overdiagnosis, n=116; confirmed CMA, n=16). Histogram C (CMA overdiagnosis, n=68 ; confirmed CMA, n=10) compares the time delay (days) between when symptoms relevant for diagnosis first started and diagnosis of reaction to cow’s milk (diagnostic delay). Number of participants differ due to missing information for different variables. A Mann-Whitney U test was undertaken to determine differences between the CMA overdiagnosis and confirmed CMA group for age at symptom onset, age at diagnosis or diagnostic delay. No statistically significant difference was found.

# Figure S3. Timing of dietary interventions for possible cow’s milk hypersensitivity

**(A)**  **(B)**


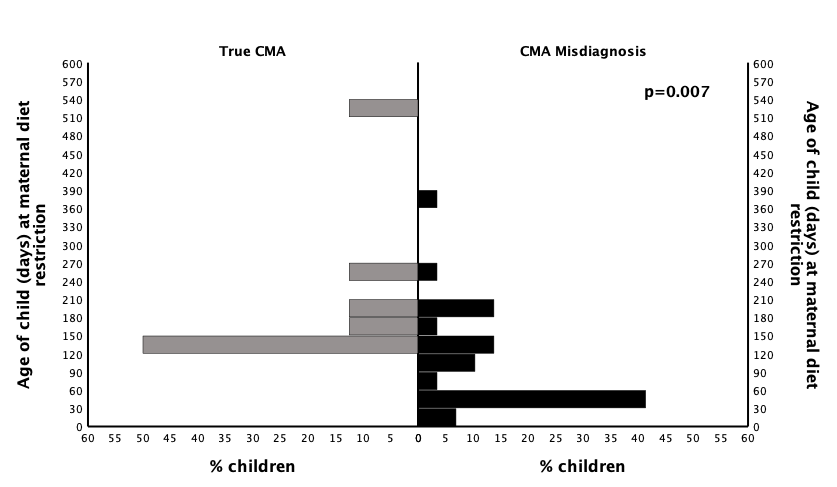

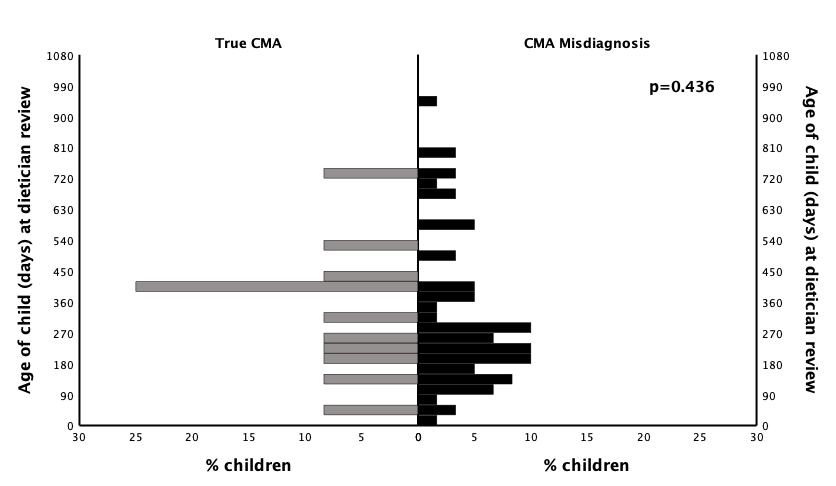


Histograms showing (A) age of child (days) when maternal dietary restriction was undertaken or advised (CMA overdiagnosis, n=29; confirmed CMA, n=8) and (B) age of child at time of Dietician review (CMA overdiagnosis, n=60; confirmed CMA, n=12). One BEEP participant without confirmed milk allergy attended a Dietician at 5 years old. Number of participants differ due to missing information for different variables. A Mann-Whitney U test was undertaken to determine differences between CMA overdiagnosis and confirmed CMA for maternal dietary restriction and dietician review. There was a significant difference in timing of maternal dietary restriction, but not for timing of dietician review.

# Figure S4. Timing of low-allergy formula prescription for possible cow’s milk hypersensitivity

**(A) (B)**


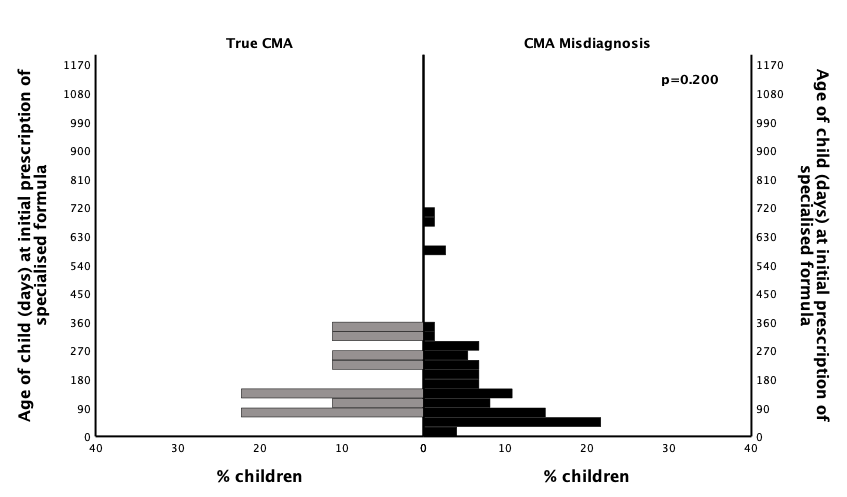

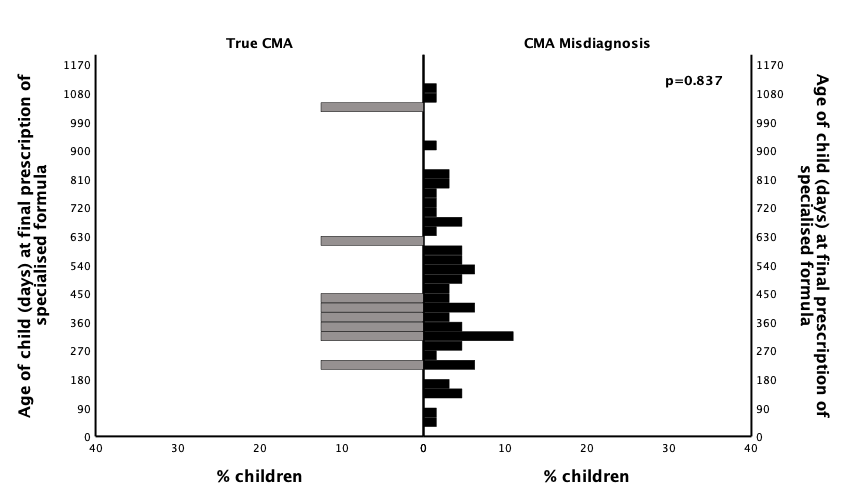


**(C)**


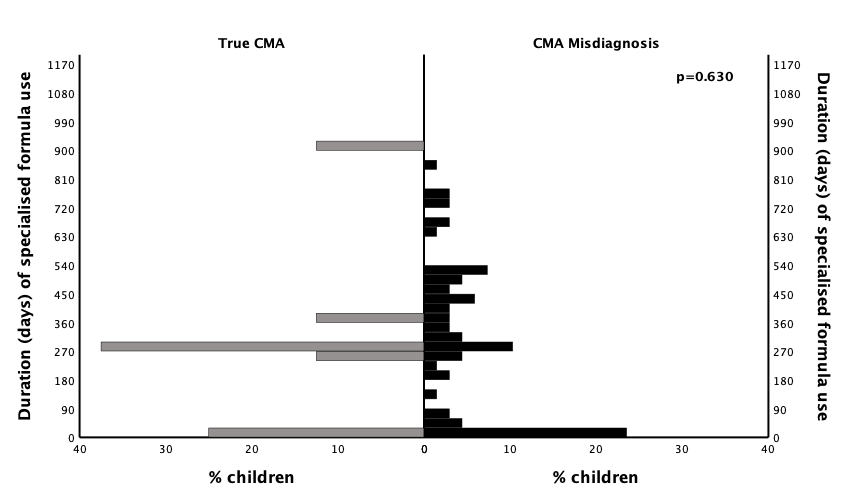


Histograms compare the age of child (days) at (A) first prescription (CMA overdiagnosis, n=74; confirmed CMA, n=9) and (B) final prescription (CMA overdiagnosis, n= 64; confirmed CMA, n=8) of low-allergy formula. Two participants without confirmed milk allergy received their final prescription of low-allergy formula at age 5 years. Number of participants differ due to missing information for different variables. Low-allergy formula refers to extensively hydrolysed (ehf), amino acid (aaf) and soya formula and excludes products designed for over 1 year old. Histogram C (CMA overdiagnosis, n=68; confirmed CMA, n=8) compares the time (days) from initial to final prescription of low-allergy formula (duration of low-allergy formula use). For those participants with missing age at final prescription (n=4) and those with a single prescription only (n=14), duration of low-allergy formula use was estimated according to volume of low-allergy formula prescribed and expected daily infant formula consumption of 780ml for infants aged <180 days old, 600ml for >/=180 days old at time of prescription of formula. ^33^ A Mann-Whitney U test was undertaken to determine differences between CMA overdiagnosis and confirmed CMA group for age at first and final prescription, and duration of prescribed formula. No statistically significant differences were found.

# Figure S5. Timing of extensively hydrolysed and amino acid formula prescription for possible cow’s milk hypersensitivity

**(A)**   **(B)**


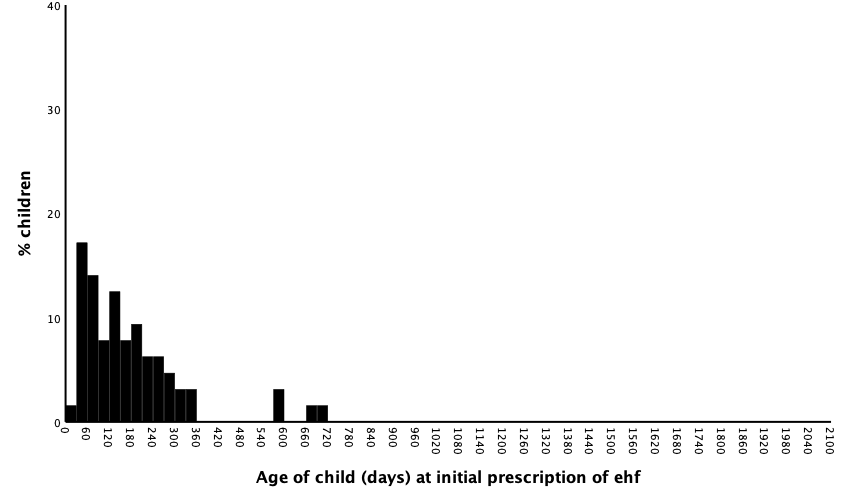

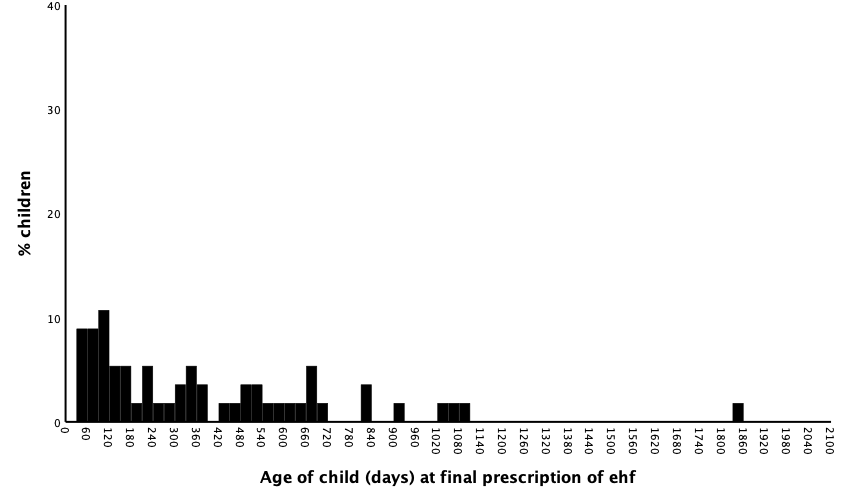


**(C) (D)**


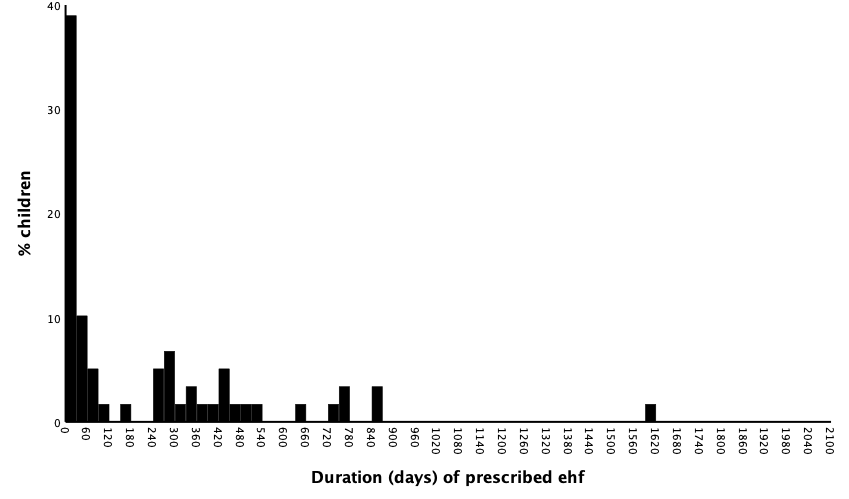

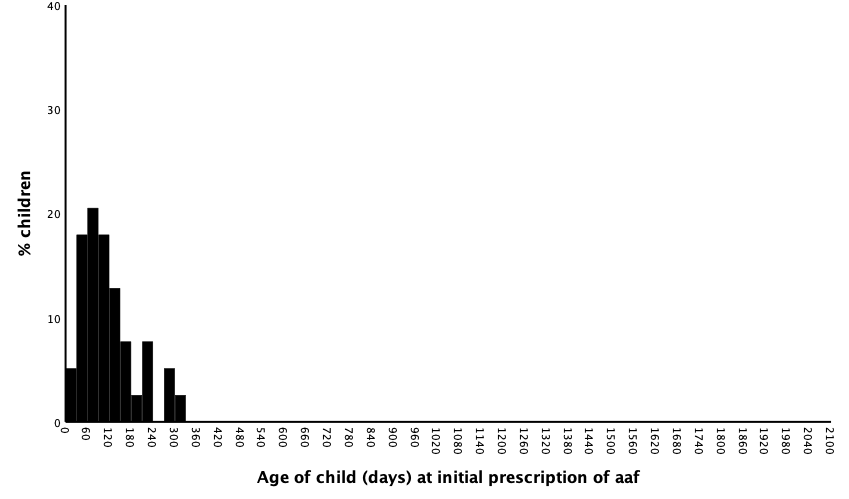


**(E) (F)**


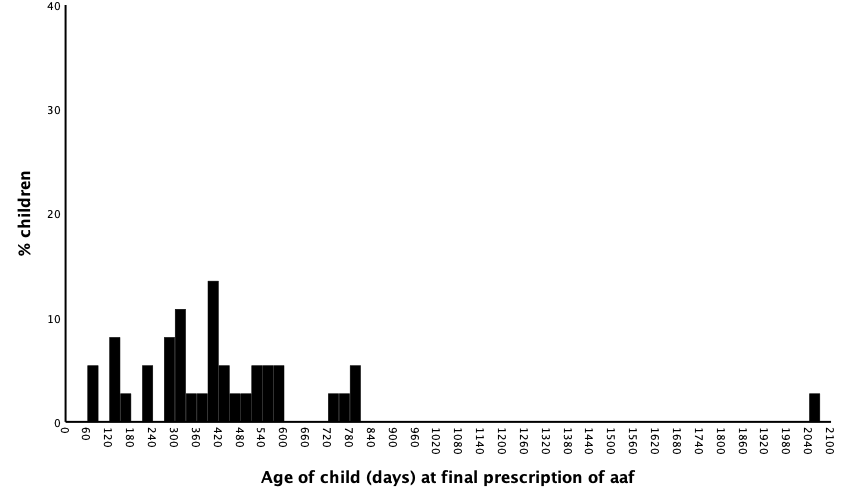

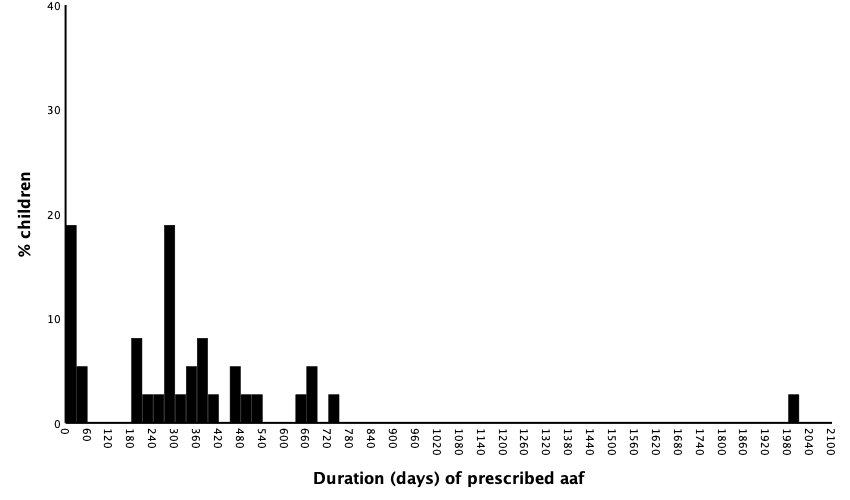


Histograms showing age at initial prescription of ehf (n=64), final prescription of ehf (n=56), duration (days) of ehf (n=59), age at initial prescription of aaf (n=39), final prescription of aaf (n=37) and duration (days) of aaf (n=37). Products designed for age >1 year old were excluded from analysis. Participants with confirmed CMA and CMA overdiagnosis were included in analysis. For those participants with missing age at final prescription, duration was calculated according to volume of low-allergy formula prescribed and expected daily infant formula consumption of 780ml for infants aged <180 days old, 600ml for >/=180 days old at time of prescription of formula. (8)

Figure S6. Symptoms recorded at time of first reaction to milk in children with confirmed CMA


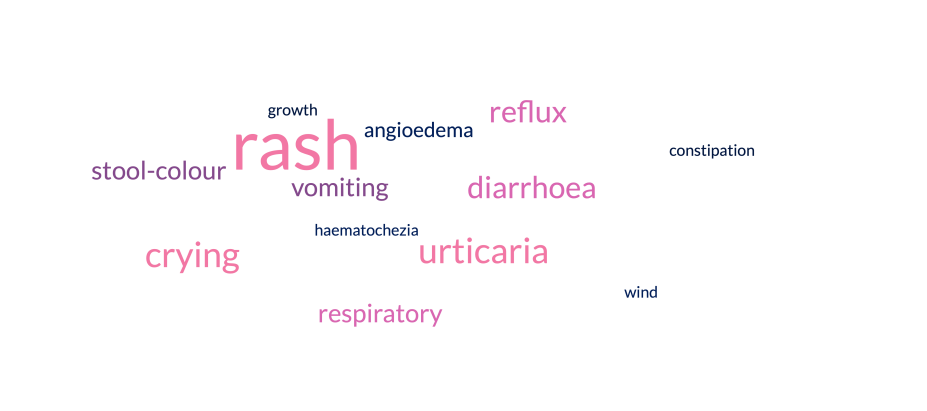


Symptoms recorded in the primary care record at time of first mention of a reaction to cow’s milk in children who had confirmed CMA. Size of words represents the frequency of individual symptoms leading to a diagnosis of possible milk reaction. Word cloud was generated using [https://www.freewordcloudgenerator.com.](https://www.freewordcloudgenerator.com./)

# References

(1) Chalmers JR, Haines RH, Bradshaw LE, et al. Daily emollient during infancy for prevention of eczema: The BEEP randomised controlled trial. *Lancet*. 2020;395(10228):962-972. doi: 10.1016/S0140-6736(19)32984-8.

(2) Kelleher MM, Jay N, Perkin MR, et al. An algorithm for diagnosing IgE‐mediated food allergy in study participants who do not undergo food challenge. *Clin Exp Allergy.* 2020 Mar;50(3):334-342. doi: 10.1111/cea.13577.

(3) Sterne JA, White IR, Carlin JB, et al. Multiple imputation for missing data in epidemiological and clinical research: potential and pitfalls. *BMJ.* 2009; 338 (b2393): 10.1136/bmj.b2393.

(4) Knol MJ, Janssen KJM, Donders ART, Egberts ACG, Heerdink ER, Grobbee DE, et al. Unpredictable bias when using the missing indicator method or complete case analysis for missing confounder values: an empirical example. *J Clin Epidemiol.* 2010; 63 (7): 728-736. 10.1016/j.jclinepi.2009.08.028.

(5) White IR, Royston P, Wood AM. Multiple imputation using chained equations: Issues and guidance for practice. *Statistics in medicine.* 2011; 30 (4): 377-399. 10.1002/sim.4067.

(6) Graham JW, Olchowski AE, Gilreath TD. How Many Imputations are Really Needed? Some Practical Clarifications of Multiple Imputation Theory. *Prevention science.* 2007; 8 (3): 206-213. 10.1007/s11121-007-0070-9.

(7) Allen HI, Pendower U, Santer M, et al. Detection and management of milk allergy: Delphi consensus study. *Clin Exp Allergy*. 2022 Jul;52(7):848-858. doi: 10.1111/cea.14179.

(8) Grimes CA, Szymlek-Gay E, Nicklas TA. Beverage consumption among U.S. children aged 0–24 months: National health and nutrition examination survey (NHANES). *Nutrients.* 2017; 9 (3): 264.

(9) Mehta S, Allen HI, Campbell DE, et al. Trends in use of specialised formula for managing cow's milk allergy in young children. *Clin Exp Allergy.* 2022 Jul;52(7):839-847. doi: 10.1111/cea.14180.

(10) Office for health improvement and disparities, UK Government. *Feeding young children aged 1 to 5 years - summary report.* 2023.

(11) Office of National Statistics. *Pregnancy and ethnic factors influencing births and infant mortality: England.* <https://www.ons.gov.uk/peoplepopulationandcommunity/healthandsocialcare/causesofdeath/datasets/pregnancyandethnicfactorsinfluencingbirthsandinfantmortalityengland> [Accessed 6th October 2023].

(12) NHS Digital. *NHS maternity statistics 2013-2015.* <https://digital.nhs.uk/data-and-information/publications/statistical/nhs-maternity-statistics/2013-15> [Accessed 6th October 2023].

(13) NHS England. *Table 1: Initiation of breastfeeding, England trend. 2013/2014.* <https://www.england.nhs.uk/statistics/statistical-work-areas/maternity-and-breastfeeding/> [Accessed 6th October 2023].

(14) Brown SJ, Relton CL, Liao H, et al. Filaggrin null mutations and childhood atopic eczema: A population-based case-control study. *J Allergy Clin Immunol.* 2008; 121 (4): 940-946.e3. 10.1016/j.jaci.2008.01.013.
